# Systematic Isolation and Characterization of Cadmium Tolerant Genes in Tobacco: A cDNA Library Construction and Screening Approach

**DOI:** 10.1371/journal.pone.0161147

**Published:** 2016-08-31

**Authors:** Mei Zhang, Hui Mo, Wen Sun, Yan Guo, Jing Li

**Affiliations:** 1 Key Laboratory of Plant Resources Conservation and Sustainable Utilization, South China Botanical Garden, Chinese Academy of Sciences, Guangzhou, 510650, China; 2 University of the Chinese Academy of Sciences, Beijing, 100039, China; Institute of Genetics and Developmental Biology Chinese Academy of Sciences, CHINA

## Abstract

Heavy metal pollution is a major limiting factor that severely affects plant growth worldwide, and the accumulation of heavy metal in the plant may be hazardous to human health. To identify the processes involved in cadmium detoxification, we constructed a cDNA library of tobacco roots acclimated to cadmium (Cd) stress. According to the results of functional screening cDNA library with a yeast Cd-sensitive mutant, *ycf1Δ*, we obtained a series of candidate genes that were involved in Cd response. Sequence analysis and yeast functional complementation of 24 positive cDNA clones revealed that, in addition to antioxidant genes, genes implicated in abiotic and biotic stress defenses, cellular metabolism, and signal transduction showed Cd detoxification effects in yeast. The real time RT-PCR analyses revealed that some Cd tolerance/ detoxification genes may be able to anticipate in other stresses such as biotic defense and water balance in tobacco. Taken together, our data suggest that plants’ acclimation to Cd stress is a highly complex process associated with broad gene functions. Moreover, our results provide insights into the Cd detoxification mechanisms along with the antioxidant system, defense gene induction, and calcium signal pathway.

## Introduction

Plant growth requires different essential micronutrients, including heavy metals, such as Zn, Mn, Ni and Cu, for normal functioning of cellular metabolism. However, in nature, there are some heavy metal elements (such as Cd, Hg and Pb), which are non-essential for plant growth. Even at trace levels in environment, these non-essential metals may cause substantial damage to organisms. Cadmium, usually as a mimic of Zn [1], is a severe environmental pollutant with high toxicity to organisms [2]. With the development of industry and modern agriculture, the environmental pollution has become increasingly serious. The active free Cd ion is supplied to soil, air, and water mainly by electroplating, battery, alloy manufacturing, pigment, and mining processes, or by fertilization of land with phosphate or sewage sludge. Cadmium is easily taken up by plant roots and leaves, and then transferred from plants into the human body [3, 4]. In general, besides the main absorption by food chain from rice or vegetables, tobacco is another main source of Cd accumulation in human body, especially for smoking population [5].

Tobacco is one of the Cd accumulating and tolerant plants, and heavy metal deposition in tobacco leaves had profound impact on tobacco industry in China [6]. A statistical analysis reported by the Chinese government in 2014 showed that 19.4% of the total cultivated land has been polluted, and the most serious pollutant is Cd [7]. To address this issue, phytoremediation, an integration biotechnology combining botany with ecology, has received substantial attention for a long time. Phytoremediation is mainly defined by using heavy metal hyperaccumulator plant species to actively absorb the metal pollutants from environment, and thus to clean the soil [8]. Accordingly, tobacco is grown quickly, and is also a Cd accumulator with a large biomass [9], as well as a model plant in transgenic research. Therefore, development of transgenic tobacco plants with high level of Cd accumulation and tolerance tailored for remediation will further enhance the feasibility of the application of tobacco plants to phytoremediation. In general, through the isolation and characterization of Cd-responding or detoxification genes in tobacco, we can explore in depth the molecular mechanisms of Cd absorption or accumulation in the plant. We then apply these functional genes by performing a genetic modification study, which results in the heavy metal content change in tobacco or other crops.

In this study, we reported the construction and functional screening of tobacco roots’ cDNA library with the yeast Cd-sensitive mutant, *ycf1Δ*, and identified additional novel factors involved in Cd homeostasis and detoxification. Among the clones isolated, we picked 24 novel genes encoding metallothioneins (MTs), transporters, cysteine-rich peptides, glycine-rich protein, calcium-binding protein, and other uncharacterized proteins. Our results provide new insights into the basic mechanisms of tobacco responding to Cd and elucidate the physiological roles for antioxidant systems in metal detoxification/accumulation, which might contribute to generating Cd low-accumulation or tolerant tobacco. It may also be crucial for improving the plant performances in phytoextraction of Cd from environment.

## Materials and Methods

### Plant materials, growth conditions and stress treatments

*Nicotiana tabacum* (*L*.) cv. SR1 seeds were germinated in half-strength Murashige and Skoog stock (MS) solid medium with 0.8% agar within a growth chamber under controlled environmental conditions with a 14 h light/10 h dark cycle (light intensity of 200 mmol m^−2^ s^−1^), a 25/20°C light/dark temperature regime and 60% relative humidity. The tobacco seedlings were then transferred into an aerated hydroponic culture system for further growing with a change of full-strength nutrient solution every 48 h. Subsequently, Cd stress (50 μM CdCl_2_), MeJA (90 μM), methyl viologen (100 μM) and Mannitol (300 mM) was imposed by supplying a nutrient solution with the corresponding chemicals for 24 h. To construct the cDNA library, the tobacco roots were harvested 24 h after the Cd treatment; for real time RT-PCR, the tobacco leaves and roots were harvested at 0 h (control), 6 h and 24 h. Experiments were performed following a completely randomized design with three replications and repeated at least twice.

### Assessment of hydrogen peroxide (H_2_O_2_) content and water loss rate in tobacco leaves

For H_2_O_2_ content detection, the tobacco seedlings with or without Cd stress were ground in acetone at room temperature. The H_2_O_2_ content was determined using the method reported by Hu et al. [10]. In brief, 1 ml of the above supernatant was mixed with 1 ml of 0.1% titanium sulfate in 20% H_2_SO_4_ (v/v) thoroughly for 10 min. After centrifuging at 12000 rpm for 10 min at room temperature, the absorbance of the supernatant was measured at 410 nm using the known concentration of H_2_O_2_ as a standard.

For comparing the water loss rate of tobacco leaves, about twenty leaves were punched (1 cm in diam.) from tobacco seedling plants under Cd stress at different times (0, 6 and 24 h). The punched leaf discs were weighed, and water loss rate was then calculated from the control tobacco leaves (CK, 0 h), with the weight of the leaves determined at each time point.

### RNA Isolation and cDNA library construction

Total RNA was extracted with Trizol Reagent (Invitrogen) and Poly (A)^+^ RNA was isolated from the total RNA using the FastTrack^®^ MAG mRNA Isolation Kit (Invitrogen, Thermo Fisher Scientific Inc.). The mRNA yield and quality were determined by spectrophotometry at 260 and 280 nm. Then total mRNA was reversely transcribed to cDNA with SMART cDNA Synthesis Kit (Clontech) according to the manufacturer’s instructions. The ds-cDNA was cloned into the pDONR222 vector using BP reaction of the Gateway cloning technology, and the entry clone library was obtained. Using the LR reaction of the Gateway cloning technology, the yeast express vector pYES-DEST52 was ligated with the entry clone to generate the secondary yeast expression library, in which the total tobacco root cDNA were inserted into the yeast expression cassata under the galactose induced promoter pGAL1.

### Yeast mutant strains and library screening using *ycf1Δ*

The yeast (*Saccharomyces cerevisiae*) mutant *ycf1Δ* (BY4741; *MATa*; *ura3Δ0*; *leu2Δ0*; *his3Δ1*; *met15Δ0*; *YDR135c*::*kanMX4*; Y04069), *yap1Δ* (BY4741; *MATa*; *ura3Δ0*; *leu2Δ0*; *his3Δ1*; *met15Δ0*; *YML007w*::*kanMX4*; Y00569), *skn7Δ* (BY4741; *MATa*; *ura3Δ0*; *leu2Δ0*; *his3Δ1*; *met15Δ0*; *YHR206w*::*kanMX4*; Y02900) and their isogenic wild-type BY4741 (*MATa*; *his3Δ1*; *leu2Δ0*; *met15Δ0*; *ura3Δ0*, Y00000) were obtained from Euroscarf (http://www.uni-frankfurt.de/fb15/mikro/euroscarf/). The yeast mutant strain *ycf1Δ* is hypersensitive to CdCl_2_ because of the deletion of its *YCF1* (*yeast cadmium factor 1*, encoding an ATP-binding cassette glutathione S-conjugate transporter) [11]. The strains identified as *yap1Δ* and *skn7Δ* are H_2_O_2_-sensitive yeast mutant strains, with deleted transcription factors (*YAP1* and *SKN7*, respectively) that are involved in oxidative stress response [12].

A tobacco root cDNA library constructed in pYES-DEST52 was introduced into *ycf1Δ* according to the method of Gietz and Woods [13]. Transformants were selected on plates containing a 2% galactose-containing synthetic drop-out (SD) medium lacking uracil (SD-Ura) plus 100 μM CdCl_2_ and grown for 5–10 days. The plasmids from the surviving yeast clones were rescued in *Escherichia coli* and extracted. Ninety-seven plasmids were selected and sequenced with an automatic sequencing machine (ABI, Columbia, MD).

### Construction of yeast expression recombinant plasmid

The isolated cDNA clones encoding Cd-tolerant candidate genes in tobacco, were reconstructed into yeast express vector, pYES260, with the exact ORF (open reading frame) of cDNA, without 3’ or 5’ UTR (untranslational region) sequences. The ORFs were amplified with the primers shown in S1 Table. The PCR fragments were subsequently inserted into the *Nco*I and *Bam*HI sites of pYES260 (Euroscarf, P30084), following the GAL1 promoter with the in-fusion technique (BD In-Fusion PCR cloning Kit, Takara Bio USA), yielding recombinant plasmids. The recombinant plasmids and pYES260 were then transformed into yeast cells, following Gietz and Woods [13].

### Cadmium and H_2_O_2_ sensitivity assays in yeast

Yeast transformants were pre-cultured in SD-Ura medium (plus 2% galactose) overnight at 30°C, diluted with fresh pre-warmed SD-Ura medium (plus 2% galactose), and then incubated with vigorous shaking at 30°C to reach an optical density of just over 0.5 at OD_600_. The cells were harvested by centrifugation, re-suspended in SD-Ura medium to an OD_600_ of 2, serially diluted in 10-fold steps, and 2 μl aliquots of each were finally spotted onto SD-Ura agar medium with or without Cd/H_2_O_2_. The Cd concentration used was 75 μM CdCl_2_ for the Cd-tolerant revalidation assay, and the H_2_O_2_ concentrations were 0.5 mM and 0.75 mM for the antioxidation assay. The test plates were incubated at 30°C for 2–7 days.

### Measurement of heavy metal content in yeast

Yeast cells were allowed to grow for 24 h in 2 ml of the culturing medium (SD medium plus 2% galactose) and the OD value was measured until the OD_600_ was 0.5. The yeast cultures were then diluted as 1:100 with fresh medium. Under liquid-shaking culture for about 24 h (OD_600_ till 1.0), CdCl_2_ was supplied until the Cd final concentration reached 15 μM, and yeast cells were allowed to grow for another 24 h. Then cells were collected and washed by centrifugation with ddH_2_O three times. Cell pellets were dried in the oven at 65°C for 3–5 days, and then the dried yeast piece was digested in 6 ml of nitric acid at 80°C for 1 h following the instructions of the Microwave Reaction System (Multiwave 3000, Anton Paar). After digestion, samples were diluted to 100 ml with deionized water and then analyzed with a Perkin-Elmer inductively coupled plasma (ICP) atomic absorption spectrometer (AAS).

### Real time RT-PCR analysis

Total RNA was isolated with the HiPure Plant RNA Kits (Magen) according to the manufacturer’s instructions. After concentration detection by ultraviolet spectrophotometer, first-strand cDNA was synthesized by using TransScript One-Step gDNA Removal and cDNA Synthesis SuperMix (TransGen Biotech) from 1 μg total RNA according to the manufacturer’s instructions. Real time RT-PCR was conducted using a model 7500 real-time PCR system (Applied Biosystems). To normalize the amount of total cDNA present in each reaction, the tobacco *L25 ribosomal protein* (NCBI accession number: L18908) gene was used as an internal control according to Schmidt and Delaney [14]. Real-time RT-PCR was performed in a total volume of 20 μl reaction mixtures with gene-specific primers (S2 Table). Each reaction included 10 μl of SYBR green real-time PCR master mix (Bio labs), 0.5 μM (each) forward and reverse primers, and 2 μl of cDNA template (equivalent to 100 ng cDNA). The amplification was performed using the following cycle parameters: 94°C for 30 s, followed by 40 cycles of 94°C for 15 s, and 60°C for 40 s for plate reading. Three independent biological repeats were performed for each treatment. Gene expression levels were calculated from the threshold cycle (CT) according to the 2^−ΔΔCT^ method.

### Statistical analysis

All experiments were repeated at least three times in independent experiments, and the leaf samples of independent experiments were harvested from at least five seedlings per treatment. In this research, data analysis was performed using statistical tools (Student's *t*-test) of Microsoft Excel software.

## Results

### Cadmium caused ROS accumulation and dehydration in tobacco leaves

It is known that Cd can cause a series of toxic effects on plant growth [15]. In our previous study, we found that Cd treatment could lead to leaves yellowing and wilting in tobacco seedlings, and the tobacco plants’ height and the roots’ length was restrained. In the current research, we examined the H_2_O_2_ content and water loss rate of tobacco seedlings under Cd stress.

**Fig 1:**
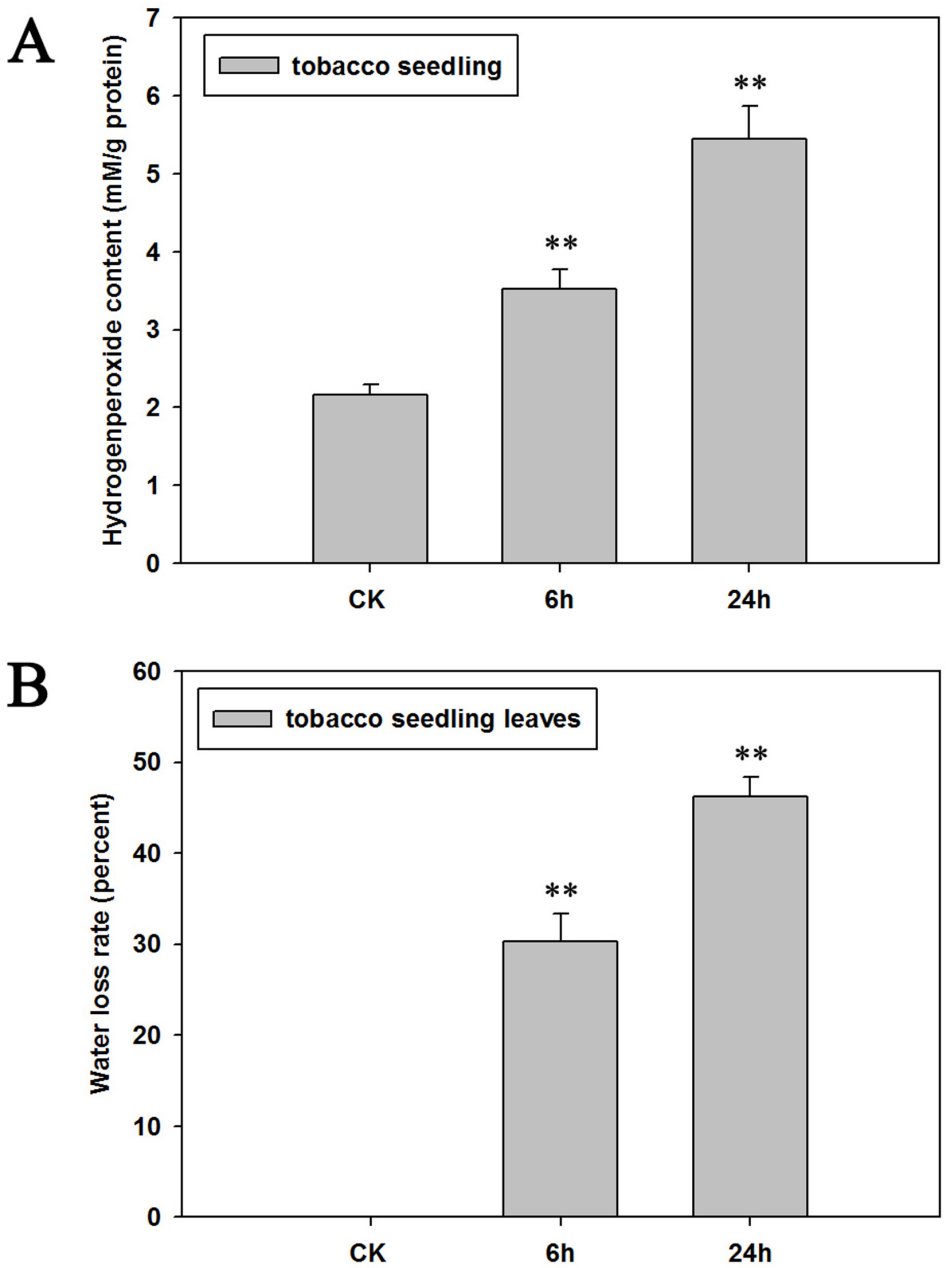
**A: H**_**2**_**O**_**2**_ **level in tobacco (SR1) seedlings under Cd stress (50 μM CdCl**_**2**_**) at different time points.** Results represent means ± standard error of four biological replicates. Double asterisk symbols (**) indicate significant differences between control (CK) and other Cd stress treatments at an individual time point (*P* < 0.01). **B: Analysis of leaf water loss (% change in leaf FW) in tobacco seedling leaves under Cd stress (50 μM CdCl**_**2**_**) at different time point.** Results represent means ± standard error of twenty biological replicates. Double asterisk symbols (**) indicate significant differences between control (CK) and other Cd stress treatments at an individual time point (*P* < 0.01).

Fig 1A shows that the H_2_O_2_ content in the Cd treated tobacco seedlings (after 6h) was much higher than the control (untreated tobacco seedlings), and the content of H_2_O_2_ increased with processing time (Fig 1A). This could be related to the ROS accumulation in tobacco seedlings in response to the Cd toxicity. Furthermore, we examined the leaf water loss of tobacco under Cd stress, and our results showed that the phenotypic characteristics of Cd treatment had a similar effect to the dehydration caused by hypertonicity. This indicates that the water loss rate of tobacco leaves increased rapidly after Cd stress (Fig 1B).

### Functional screening of tobacco root cDNA library with Cd sensitive yeast mutant

**Fig 2:**
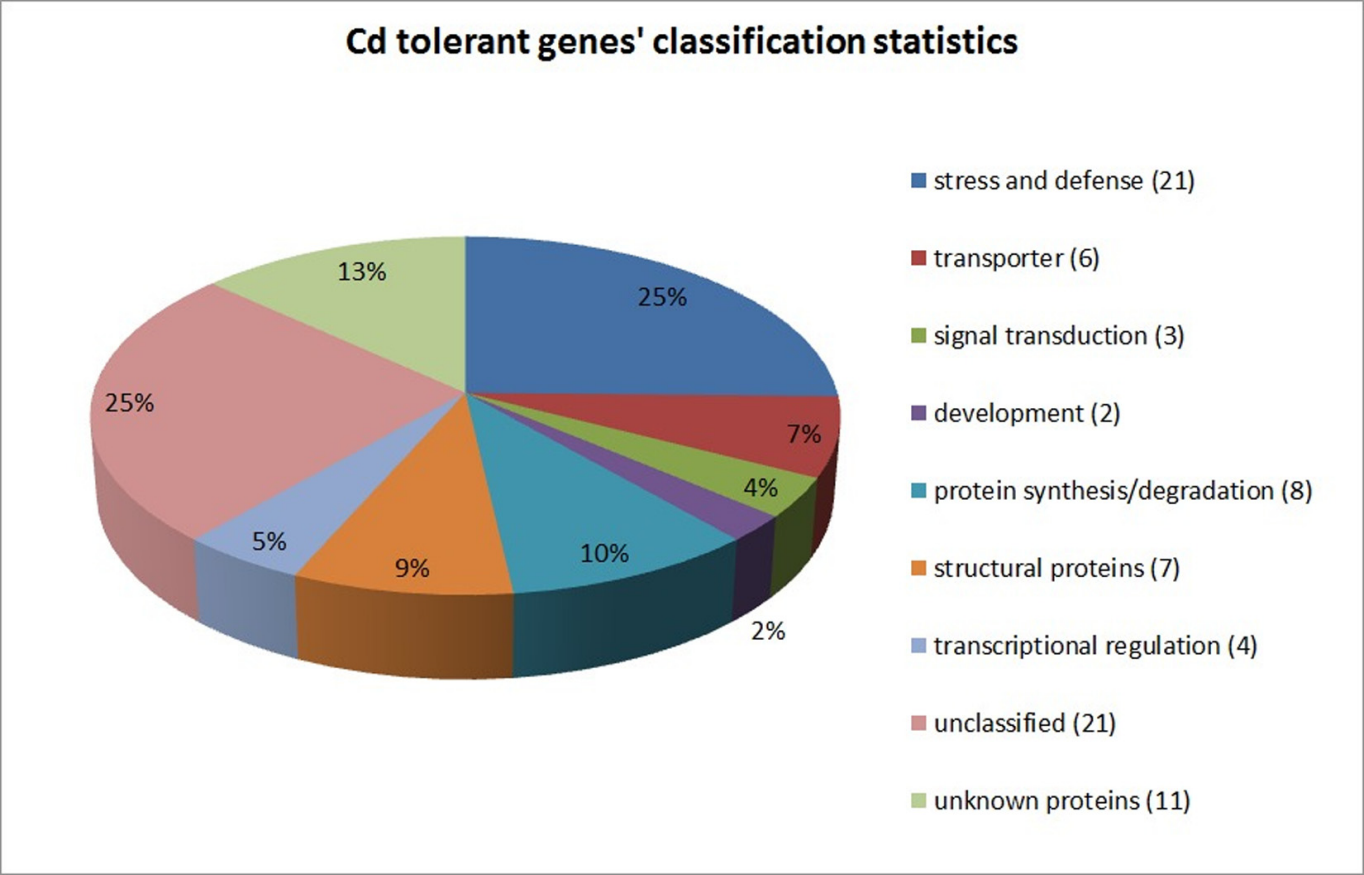
Cd tolerant genes’ classification statistics among nine functional categories. The percentage proportion (out of the total number of genes) of genes belonging to a particular functional group is shown.

The cDNA library was constructed using the Gateway technique with the roots of Cd treated tobacco seedlings, since the Cd toxicity has a direct impact on the roots. After the screening procedure with the Cd sensitive mutant *ycf1Δ*, we obtained 135 single yeast clones in total, which can grow on Cd plus SD-Ura medium (100 μM CdCl_2_, 2% galactose). These yeast clones were picked up and an amplifying culture was performed, then the obtained plasmids were identified by alkali splitting and sequenced. Finally, 107 recombinant plasmids with tobacco cDNA insertions were obtained and analyzed in the next step. After deleting some non-coded RNA sequences and poor-quality or non-referenced sequences, we ultimately acquired 97 meaningful reference recombinant clones, encoding 83 different tobacco genes (S3 Table), including 46 full-length cDNA, and 37 partial cDNA sequences. Among the 83 clones that corresponded to tobacco cDNA, 72 corresponded to unigenes, whereas the other 11 were unknown ESTs or encoding hypothetical proteins (S3 Table). The proteins encoded by these cDNAs were classified into different categories (Fig 2), including stress and defense (21), transporters (6), signal transduction (3), development (2), protein synthesis/degradation (8), structural proteins (7), transcriptional regulation (4), unclassified (21) and unknown proteins (11).

### Confirmation of candidate Cd tolerant genes in yeast

**Fig 3:**
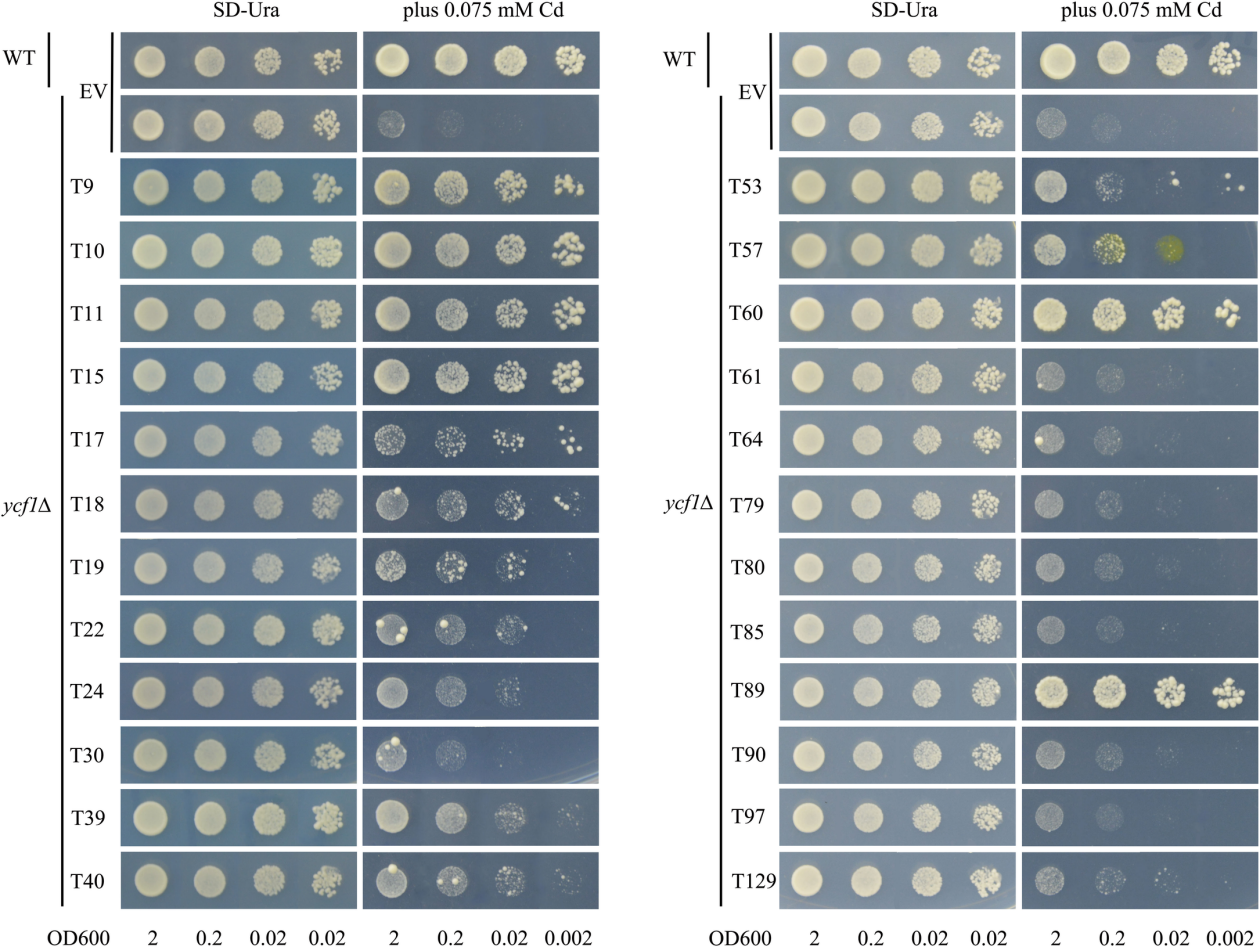
Tobacco candidate Cd detoxification genes mediate Cd tolerance in yeast. Yeast tolerance toward Cd following overexpression of *T9*, *T10*, *T11*, *T15*, *T17*, *T18*, *T19*, *T22*, *T24*, *T30*, *T39*, *T40*, *T53*, *T57*, *T60*, *T61*, *T64*, *T79*, *T80*, *T85*, *T89*, *T90*, *T97* and *T129* (24 tobacco cDNA in total) in *ycf1Δ*. The yeast cells were spotted in four concentrations (OD_600_ = 2, 0.2, 0.02, 0.002). YNB represents control culture medium with 2% galactose and without Cd added. As a negative control, the mutant strain *ycf1Δ* was transformed with the empty vector pYES260 (EV). As a positive control, wild type yeast BY4741 (WT) was transformed with the empty vector pYES260 (EV). Plates were incubated for 4 days to 1 week at 30°C.

To confirm the results of the cDNA library screening, and according to the yeast clones’ size in the screening process, we finally selected 24 full-length cDNAs to further verify the complementation effect of the yeast Cd sensitive mutant, *ycf1Δ* (Table 1). All 24 genes showed increased Cd tolerance in *ycf1Δ* to different extents (Fig 3). Among the genes, there were one *snakin-2* and five *MT* genes (*T9*, *T10*, *T11*, *T15*, *T60* and *T89*), which showed the strongest capability to tolerate Cd, followed by cDNAs encoding cysteine and the histidine-rich domain-containing protein Rar1 (*T17*), copper transporter (*T18*), an unknown protein (*T39*), and an *MT-like* protein (*T40*). We found that tobacco PLAC8 family member (*T19*), glycine-rich protein (*T22*), basic pathogenesis-related protein (*T22*), ubiquitin (*T53*), and calreticulin (*T57*) also showed a certain degree of increasing capability to tolerate the Cd, while the remaining 9 cDNAs (*T30*, *T61*, *T64*, *T79*, *T80*, *T85*, *T90*, *T97* and *T129*) can only slightly elevate the tolerance of *ycf1Δ* to 75 μM Cd. Our results further confirmed the accuracy and high efficiency of the library screening, and indicated that the application of this functional screening approach in this study was successful.

**Table 1:** Cd resistance/tolerance functional candidate cDNA list in tobacco.

Clone	GenBank match	Tolerance to Cd	Name	Blast homology	NO. ofreplications
*T9*	XM_009623044.1	++++	*Snakin-2*	Snakin-2	1
*T10*	XM_009621032.1	++++	*Metallothionein 1*	Metallothionein type 2	5
*T11*	XM_009629116.1	++++	*Metallothionein 2*	Metallothionein type 2	5
*T15*	XM_009608450.1	++++	*Metallothionein 3*	Metallothionein type 2	1
*T17*	XM_009626938.1	+++	*Rar1*	Cys and his-rich domain- containing protein RAR1	1
*T18*	XM_009801794.1	+++	*Copper transporter*	Copper transport protein CCH	1
*T19*	XM_009629259.1	++	*PLAC8 family*	Cell number regulator 8-like	1
*T22*	NM_001311181.1	++	*GRP-2*	Glycine-rich protein 2	1
*T24*	XM_009789771.1	++	*Basic PR*	Uncharacterized protein	3
*T30*	XM_009799919.1	+	*Peptidase isoform 1*	Proteasome subunit beta type-3-A	1
*T39*	XM_009759724.1	+++	*Unknown protein1*	Uncharacterized protein	1
*T40*	M97360.1	+++	*Metallothionein-like*	SAR8.2c superfamily	2
*T53*	XM_009782934.1	++	*Ubiquitin-like*	Ubiquitin-like protein 5	1
*T57*	XM_009631851.1	++	*Calreticulin*	Calreticulin	1
*T60*	XM_009765140.1	++++	*Metallothionein 4*	Metallothionein type 2	2
*T61*	XM_009795289.1	+	*CBP20*	Wound-induced protein	1
*T64*	XM_009777153.1	+	*Chitinase*	Endochitinase B	1
*T79*	XM_009791409.1	+	*GST1*	Glutathione S-transferase	1
*T80*	XM_009790972.1	+	*GST2*	Glutathione S-transferase	1
*T85*	XM_009761772.1	+	*PUB1*	E3 ubiquitin-protein ligase PUB23	1
*T89*	M97362.1	++++	*Metallothionein 5*	SAR8.2e superfamily	1
*T90*	XM_009773220.1	+	*GST3*	Glutathione S-transferase	1
*T97*	XM_009772965.1	+	*APX*	L-ascorbate peroxidase 2	1
*T129*	XM_009769585.1	+	*BPS1*	Bypass1	1

+, ++, +++, and ++++ represent the elevated Cd tolerance level in yeast mutant strain, *ycf1Δ*, show the cDNA-overexpression transgenic *ycf1Δ*’s growth observed at 1, 10^−1^, 10^−2^, 10^−3^ dilutions, respectively (see Fig 3).

**Fig 4:**
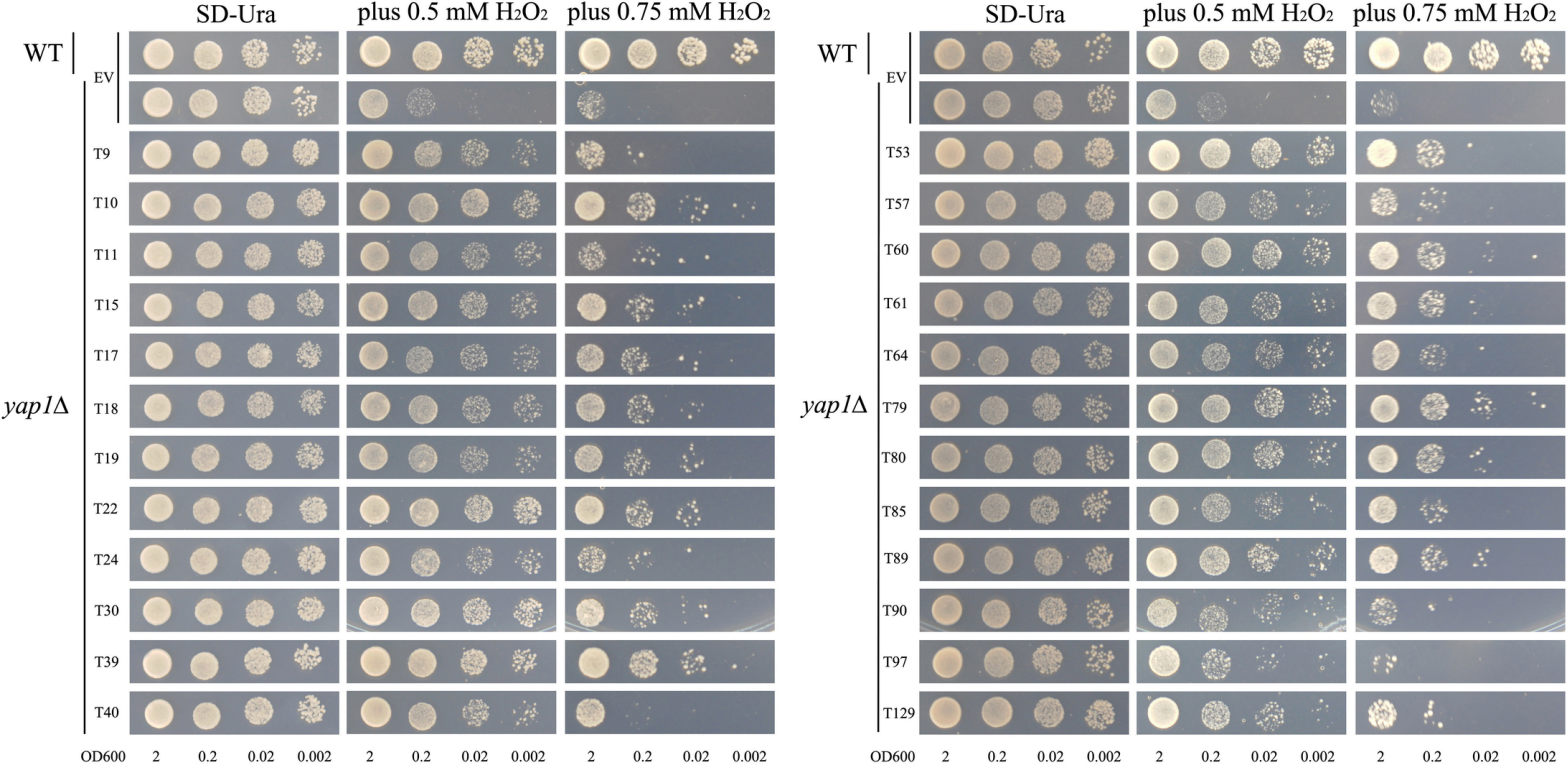
The oxidation resistance test of tobacco cDNA (24 candidate Cd detoxification cDNA in total) overexpression in yeast mutant *yap1Δ*. Yeast cultures were adjusted to OD_600_ = 2, 0.2, 0.02, 0.002 and 2 μl serial dilutions (from left to right in each panel) were spotted on SD medium without (YNB) or with different H_2_O_2_ (0.5 mM and 0.75 mM) concentrations. As a negative control, the mutant strain *yap1Δ* was transformed with the empty vector pYES260 (EV). As a positive control, wild type yeast BY4741 (WT) was transformed with the empty vector pYES260 (EV). Plates were incubated for 6 days at 30°C.

**Fig 5:**
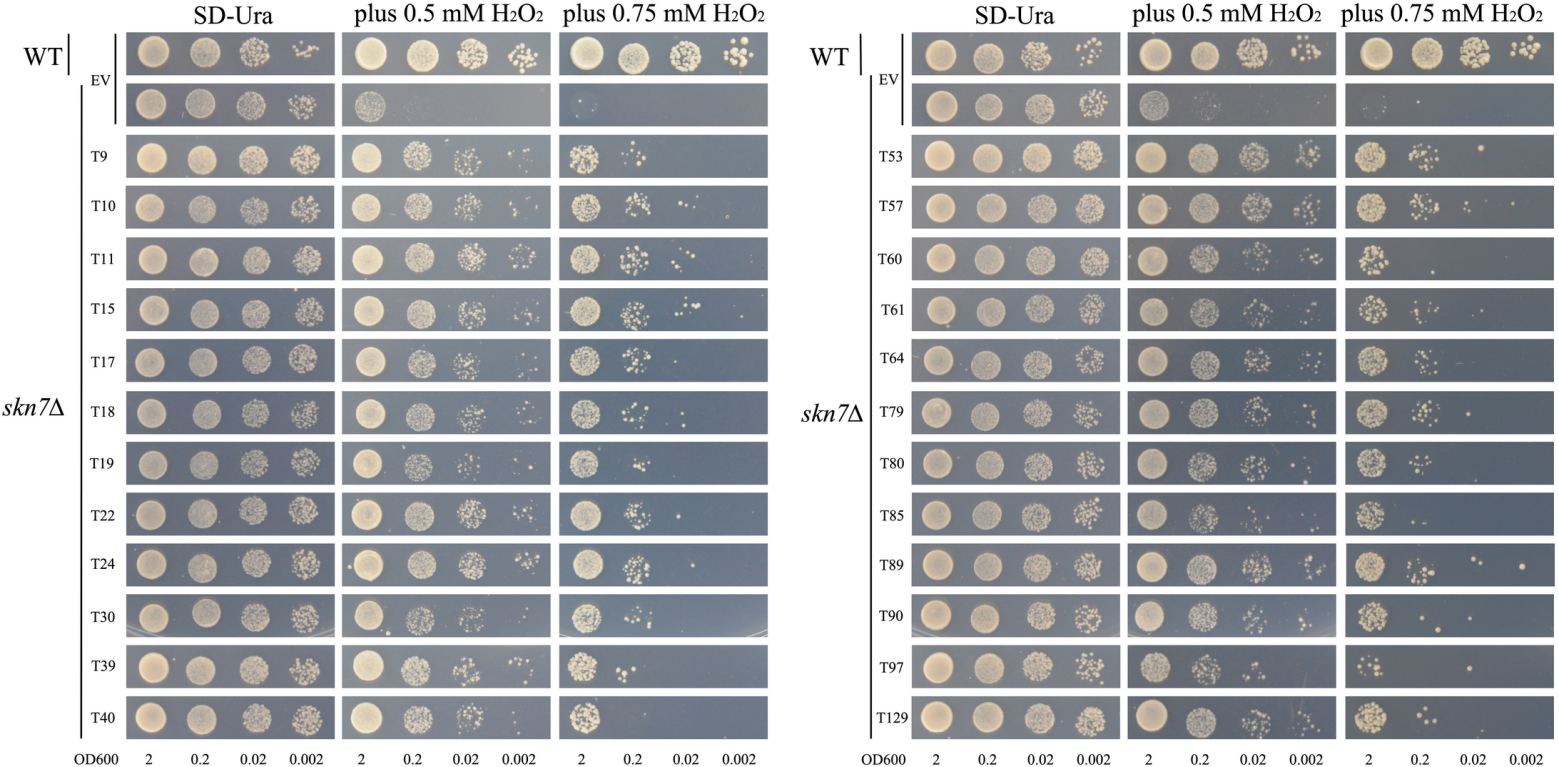
Oxidation resistance test of tobacco cDNA (24 candidate Cd detoxification cDNA in total) overexpression in yeast mutant *skn7Δ*. Yeast cultures were adjusted to OD_600_ = 2, 0.2, 0.02, 0.002 and 2 μl serial dilutions (from left to right in each panel) were spotted on SD medium without (YNB) or with different H_2_O_2_ (0.5 mM and 0.75 mM) concentrations. As a negative control, the mutant strain *skn7Δ* was transformed with the empty vector pYES260 (EV). As a positive control, wild type yeast BY4741 (WT) was transformed with the empty vector pYES260 (EV). Plates were incubated for 6 days at 30°C.

The results of cDNA sequence analyses showed that the majority of genes involved in Cd detoxification encode antioxidant proteins. In this study, we also detected the antioxidant characteristics of these candidate genes using a functional complementation assay with H_2_O_2_ sensitive mutants, *yap1Δ* and *skn7Δ*. The genes *YAP1* and *SKN7* encode two oxidative stress-related transcriptional factors in yeast, and mutations of these two genes will result in decreasing of yeast’s oxidation resistance. In addition, *yap1Δ* and *skn7Δ* are both sensitive to H_2_O_2_ and their growth was retarded by applying H_2_O_2_ to the medium. When expressing the tobacco candidate Cd-tolerant genes in these two mutant strains, the transforming yeasts increased the tolerance to H_2_O_2_. This indicates that the products encoded by these Cd-tolerant genes all have some antioxidation activities (Figs 4 and 5).

In yeast, the transcription factors yap1 and skn7 have been proved in a cellular pathway that controls the oxidative stress response [16]. Yeast mutant strains *yap1Δ* showed Cd sensitive phenotype while *skn7Δ* not. Here we also performed the 24 candidate genes’ over-expression assay in *yap1Δ* mutant, to detect whether the candidate genes can change the *yap1Δ* mutant’s tolerance to Cd. Our result showed that the 24 Cd-tolerant candidate genes can all enhance the tolerance of *yap1Δ* mutant to Cd in a similar degree (S1 Fig), which is quite different from the same assay in *ycf1Δ* (Fig 3).

### Cadmium content in yeast affected by induced exogenous gene expression

**Fig 6:**
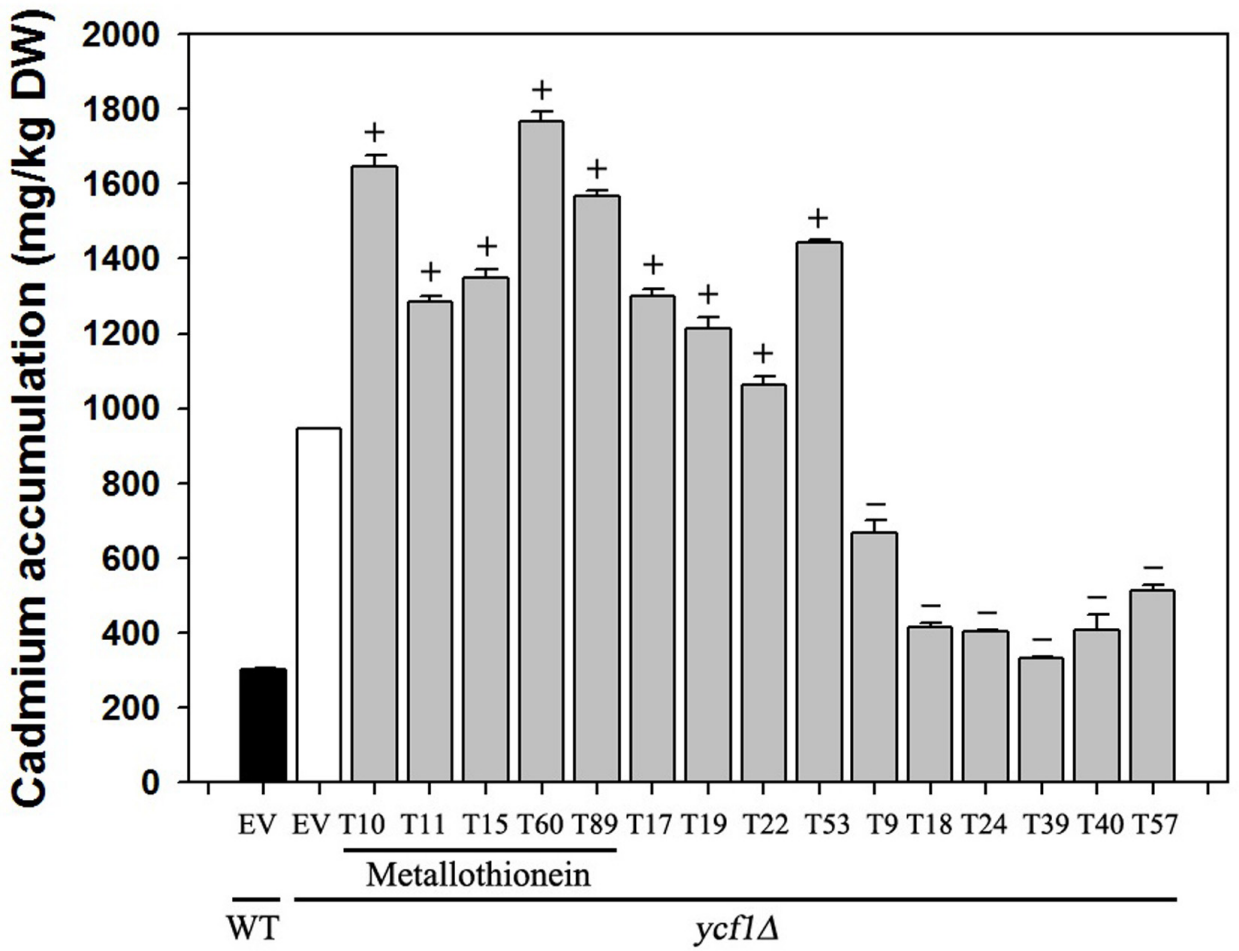
Tobacco cDNA overexpression affects Cd accumulation in yeast. 15 tobacco Cd detoxification gene cDNA (including 5 *metallothioneins*: *T10*, *T11*, *T15*, *T60*, *T89* and other cDNA: *T17*, *T19*, *T22*, *T53*, *T9*, *T18*, *T24*, *T39*, *T40*, *T57*) were overexpressed in yeast mutant *ycf1Δ*, and the Cd contents were detected using an inductively coupled plasma atomic absorption spectrometer (ICP-AAS). Here, “+” represents a Cd content that is higher than that in *ycf1Δ* expressing empty vector pYES260, “-” represents the Cd content is lower than that in *ycf1Δ* expressing empty vector pYES260 (EV), and WT represents the wild type yeast strain (BY4741). Results represent means ± standard error of three biological replicates.

Because yeast is a single-celled organism, we can deduce that the detoxification mechanisms of Cd in yeast cells can be classified into two types: chelation/sequestration and excretion, which can be reflected by the Cd accumulation in yeast. We detected the Cd content in dried transgenic yeast cells, and concluded that the possible functional mechanism of these candidate genes increase the tolerance of yeast to Cd. In this assay, we preliminarily picked out 15 tobacco cDNAs (*T9*, *T10*, *T11*, *T15*, *T17*, *T18*, *T19*, *T22*, *T24*, *T39*, *T40*, *T53*, *T57*, *T60*, *T89*) according to the yeast mutant strains complementary assay, which showed stronger capability to elevate the tolerance of *ycf1Δ* to Cd stress. The results shown in Fig 6 indicated that the overexpression of tobacco Cd-tolerant candidate genes in yeast could change the Cd content in yeast cells, and these genes may elevate *ycf1Δ* tolerance to Cd in different ways. The results of this research indicate that one part of the cDNAs (*T10*, *T11*, *T15*, *T60*, *T89*, *T17*, *T19*, *T22*, *T53*) over-expression in yeast increased the accumulation of Cd in cells, while another portion of cDNAs (*T9*, *T18*, *T24*, *T39*, *T40*, *T57*) decreased the accumulation (Fig 6). These results indicate that the Cd detoxification mechanism mediated by these two types of genes might be different in single cells (yeast); one mechanism by chelating Cd and then deactivating the Cd toxicity, while the other is by excreting the Cd.

### Expression patterns of candidate genes under Cd, methyl viologen and mannitol stresses

**Fig 7:**
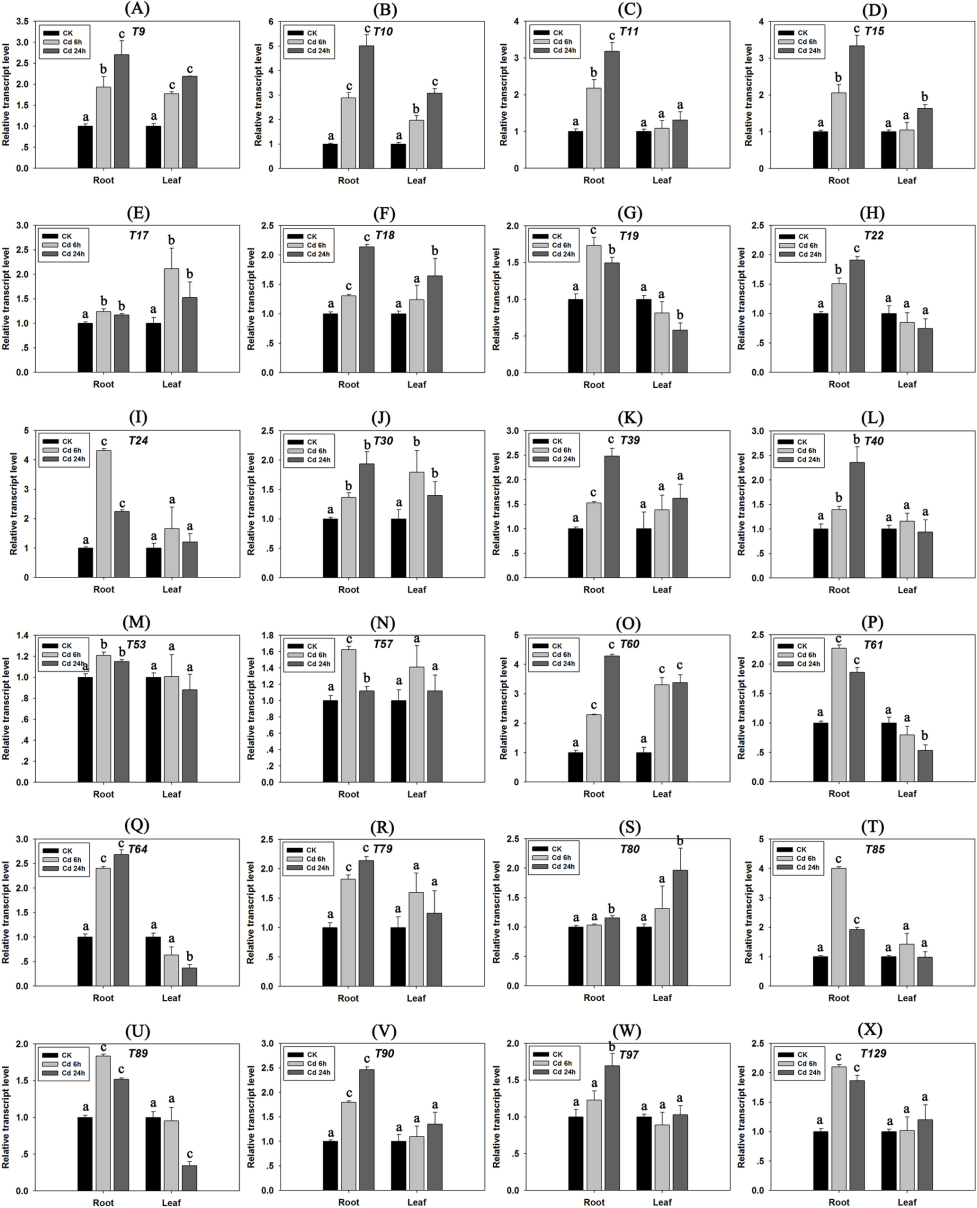
Gene expression analyses of 24 candidate Cd detoxification genes in tobacco seedling roots and leaves under Cd stress (50 μM CdCl_2_). Results represent means ± standard error of three biological replicates. Error bars: “a” indicates no significant differences (*P* > 0.05, compared with CK); “b” indicates a significant difference (0.01 < *P* < 0.05, compared with CK); and “c” indicates a highly significant difference (*P* < 0.01, compared with CK).

Quantitative expression analysis of the 24 candidate genes was performed to validate whether the exogenous Cd stress affected the gene expression. Since we can conclude that these 24 candidate genes are involved in Cd detoxification in yeast cells, if their expression in tobacco plant could be affected by Cd stress, we can infer that further these genes would indeed be involved in the Cd response of tobacco in vivo. As shown in Fig 7, most of these candidate genes’ expression could be affected by Cd stress, especially in roots. With the exception of T53 and T57, other genes’ expression could be induced by Cd above two fold, either in the root or in the leaf. This indicates that at the transcriptional level, the biological function of these genes might be involved in the Cd response.

We also checked the genes’ expression patterns under methyl viologen (oxidative stress) and mannitol (simulating dehydration) stresses by real time RT-PCR analyses, and the results showed that all the genes’ expression could be affected by methyl viologen (oxidative stress) and mannitol (simulating dehydration) stresses (S2A–S2X Fig), indicated that the Cd tolerant candidate genes might also be involved in responding to oxidative stress or dehydration in tobacco.

These genes’ expressions were normalized against tobacco *L25 ribosomal protein* rRNA to give the relative gene expression wherein error bars represent the standard error of mean (SEM) (Fig 7A–7X). The changes at the transcription level of these candidate genes under Cd, methyl viologen and mannitol stresses are further evidence that their encoding products participate in the detoxification of Cd, as well as are involved in other abiotic stresses in tobacco plants.

### The expression patterns of WRKYs, MPKs, DREBs and aquaporins under Cd stress and MeJA/Dehydration

**Fig 8:**
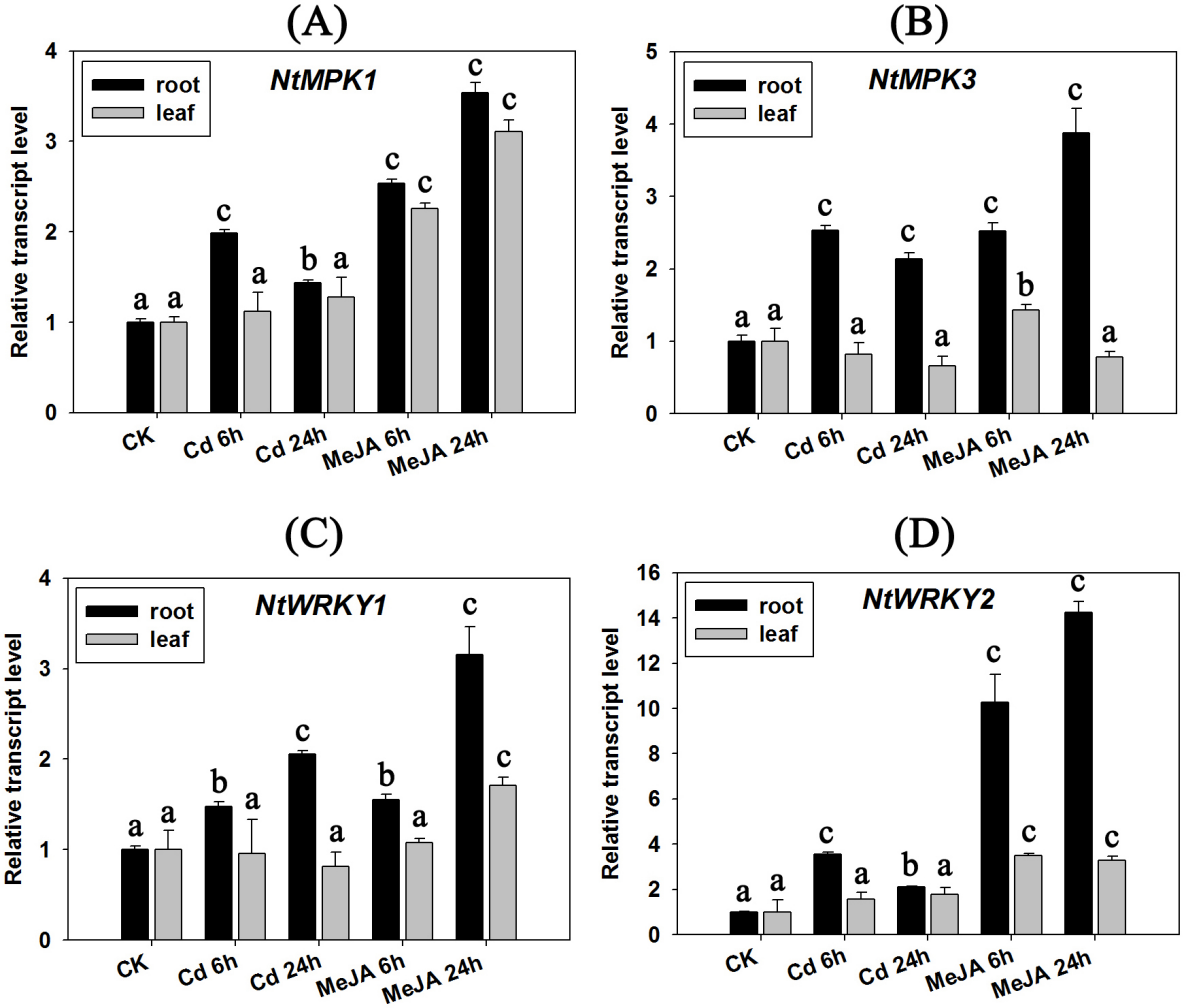
Gene expression analyses of defense-related genes under Cd stress (50 μM CdCl_2_) and MeJA treatment (90 μM, analogy of plant defense responses). A: *NtMPK1*; B: *NtMPK3*; C: *NtWRKY1*; D: *NtWRKY2*. Results represent means ± standard error of three biological replicates. Error bars: “a” indicates no significant differences (*P* > 0.05, compared with CK); “b” indicates a significant difference (0.01 < *P* < 0.05, compared with CK); and “c” indicates a highly significant difference (*P* < 0.01, compared with CK).

Table 1 and Fig 2 indicate that there are some Cd detoxification candidate genes belonging to the defense-related gene in tobacco, and there are also reports that have revealed that metal accumulation can armor plants against biotic stresses [17–19]. In our research, we chose several defense-related key regulators, including *WRKYs* (*NtWRKY1* and *NtWRKY2*) [20, 21] and *MAPKs* (*NtMPK1* and *NtMPK3*) [22], and detected their expression patterns under Cd stress and the MeJA treatment. As one of the wounding-related phytohormones and molecular signals, MeJA exists widely in plants. The exogenous application of MeJA can induce expression of plant defense-related genes, stimulate plant defense responses, then initiate the defensive system and elevate resistance to disease or pathogens. In the current research, the expression of two tobacco WRKY transcription factors (*NtWRKY1* and *NtWRKY2*) and two MAP kinases (*NtMPK1* and *NtMPK3*) was induced by Cd treatment, and they showed a similar expression pattern with the MeJA treatment (Fig 8). This indicated that tobacco WRKYs and MPKs might be involved in the Cd response by activating the down-stream defense system and inducing the expression of the defense-related genes.

**Fig 9:**
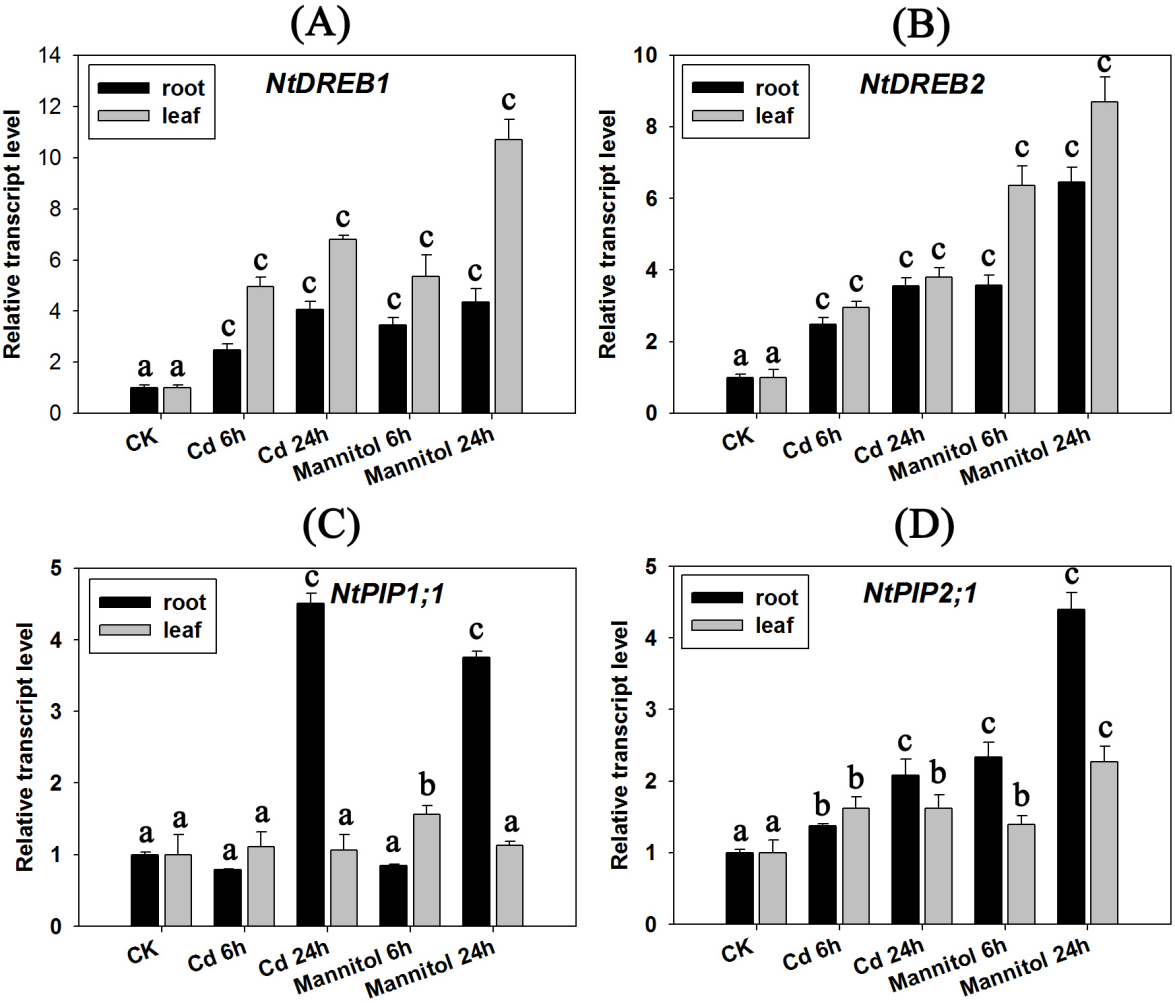
Gene expression analyses of drought-related genes under Cd stress (50 μM CdCl_2_) and mannitol treatment (300 mM, analogy of dehydration stress). A: *NtDREB1*; B: *NtDREB2*; C: *NtPIP1;1*; D: *NtPIP2;1*. Results represent means ± standard error of three biological replicates. Error bars: “a” indicates no significant differences (*P* > 0.05, compared with CK); “b” indicates a significant difference (0.01 < *P* < 0.05, compared with CK); and “c” indicates a highly significant difference (*P* < 0.01, compared with CK).

Cadmium stress can lead to disorders of the water balance in plants, and cause the wilting phenotype [23]. In the current research, we also analysed the changes to the transcript of several key regulator genes such as *d**ehydration* *r**esponsive* *e**lement* *b**inding transcription factor* (*DREBs*) and *aquaporins* under Cd and hyperosmotic stress. Plant DREBs, also known as C-repeat binding factors, (CBFs) proteins belong to APETALA2 (AP2) family transcription factors that bind to DRE/CRT cis-element and regulate the expression of stress-responsive genes, usually involved in regulation of the water balance [24]. Aquaporins are ubiquitously distributed in organisms and take an important role in maintaining water balance in the plant [25]. Fig 9 indicates that *NtDREB1* and *NtDREB2* can be clearly induced under Cd stress, and they showed similar expression patterns with mannitol treatment (hyper-osmotic stress), indicating that *NtDREB1* and *NtDREB2* are involved in the recovery of water imbalances caused by Cd toxicity. In addition, and two *aquaporins* (*NtPIP1;1* and *NtPIP2;1*) also showed an induced expression pattern under either Cd stress or mannitol treatment (Fig 9).

## Discussion

Yeast is a single-celled organism and therefore is less complex to study for initial functional screening purposes. In the current research, given the Cd-accumulating characteristic of tobacco plants, we constructed a cDNA library with tobacco roots, and screened this library by functional complementary assay with the Cd-sensitive yeast mutant strain, *ycf1Δ*, for the purpose of cloning and isolating the genes related to Cd detoxification/tolerance in tobacco. The *YCF1* gene was isolated according to its ability to confer Cd resistance from *Saccharomyces cerevisiae*, which shows extensive homology with the human *multidrug resistance-associated protein* (*MRP1*) gene [26]. The *YCF1* gene encodes an ATP-binding cassette (ABC) transporter, which functions the Cd detoxification as transmembrane pump in yeast through glutathione S-conjugated metal ion combination and translocation [11]. The results of this study demonstrated that cDNA functional screening with a yeast deficient mutant strain was an efficient approach for isolating defective-element-related candidate functional genes. In our experiment, the screening criterion for the Cd-tolerant gene was to recover the Cd-sensitive phenotype of *ycf1Δ*, which involved searching multiple pathways or mechanism processes, and obtaining genes with different degrees of Cd tolerance according to recovery levels of the yeast clones. Our results clearly showed that a series of candidate genes related to Cd tolerance screened from the cDNA library of the tobacco roots were divided into different categories according to their predicted functions (Fig 2 and S3 Table). This is different from *YCF1*’s function category: encoding a transporter protein in yeast. This result also indicated that the Cd detoxification mechanisms in vivo might be involved in diverse metabolic pathways and signal transduction.

### The Cd detoxification response in tobacco partly depends on the plant's defense system

About 100 yeast clones have been obtained according to functional screening assay with yeast mutant *ycf1Δ* and the corresponding plasmids were isolated and sequenced (S3 Table). Among them, we found a number of cDNAs encoding defense-related proteins in tobacco. *Metallothionein* (*MT*) possessed the highest emergence of probability during the library screening process (Table 1), and MTs are well-documented metal-binding proteins [27] as well as ROS scavengers [28]. In this research, we isolated five *MTs* (*T10*, *T11*, *T15*, *T60* and *T89*) and one *MT-like* (*T40*) gene, which all showed clear capabilities for complementing the *ycf1Δ* to Cd sensitive mutant trait. Although there is no current report to support that MTs were directly involved in responses to biotic stresses and defense response in plants, we found that one of tobacco *MTs* (*T89*), and *MT-like* (*T40*) belong to the SAR8.2 superfamily. This superfamily could be induced by the tobacco mosaic virus and involved in systemic acquired resistance (SAR) [29]. *Snakin-2* is also a defense-related gene and encodes a broad-spectrum antimicrobial peptide, which is involved in multiple stress responses and hormone crosstalk, in addition to maintaining the redox homeostasis in plant cells [30]. Our research indicates that *NtSnakin-2* is also a Cd detoxification gene and encodes antioxidant protein, and this is further evidence that a portion of plant defense proteins participate in heavy metal (including Cd) detoxification. Another defense related gene, tobacco *RAR1* (*T17*), encoding a cysteine and histidine-rich domain-containing protein, was also isolated in our cDNA screening approach. Tobacco RAR1 showed stronger abilities of Cd detoxification in yeast (Fig 3). Liu *et al*. [31] and Shang *et al*. [32] reported that RAR1, acting as a cochaperone of HSP90, played a key role in plant defense responses to diverse pathogens in tobacco and *Arabidopsis*. In addition, *T24* encoding a basic pathogenesis-related protein [33], also showed a strong tolerance to Cd. Furthermore, this cDNA had three replications in our library screening process, which indicates that this protein could also be an important Cd detoxification/tolerant-related protein. There were also some other identified defense-related genes, including *Glycine-rich proteins* (*T22*, *T95*) [34], *Peptidase isoform 1* (*T30*) [35], *Chitinase* (*T64*) [36], *Glutathione S-transferase* (*T79*, *T80*, *T90*) [37], *CBP20* (*T61*) [38], which seem to be involved in detoxification/tolerant in yeast by functional complementary assay (Fig 3) and in the tobacco plant by expression pattern analyses (Fig 7).

Scientists have long known that high concentrations of heavy metal in plants can increase resistance against pathogens and herbivores, and accordingly raised the defense hypothesis of elemental hyperaccumulation [17, 39]. Fones *et al*. [18] found that metal accumulation in *Thlaspi caerulescens* might induce the antimicrobial metabolites and further cause resistance during plant defense response. However, in the current research, we provided direct evidence from the angle of molecular biology that plant defense related proteins were involved in Cd detoxification, and these genes’ expression could be induced by Cd. Accordingly, we speculated that Cd-induced accumulation of defense-related proteins in tobacco might increase the resistance to biotic stresses (such as pathogens and herbivores), which proved the defense hypothesis of metals [17], and suggested Cd (or other heavy metals) might be an elicitor to induce resistance responses of plants to biotic stresses. Related studies should be performed by transgenic assay in tobacco to prove our hypothesis.

### Crosstalk between Cd stress and other biotic or abiotic stresses

Unlike plants responses to biotic stress factors, such as fungi, viruses and herbivores, heavy metal stresses, especially Cd, are not coevolution long-lasting events for plants, but cause plants to develop passive-adaptive mechanisms *in vivo*. Many reports about the toxicity of Cd to plants and its mechanisms have been reviewed [15, 40, 41]. In general, the toxic symptoms of Cd in plants include growth retardation, alterations of photosynthesis, enzyme deactivation, interferences with mineral uptake, disturbance of water balance. Of these, Cd-induced cellular oxidative stress caused by ROS (reactive oxygen species) accumulation is the most important issue. The generation of ROS is one of the most common plant responses to different stresses, representing a key cross-point at which various signaling pathways come together [42]. In this paper, with reference to other’s previous researches and combination of the phenotype of tobacco seedlings subject to Cd treatment, we mainly focus on the crosstalk between the Cd stress signal pathway and defense/dehydration stress.

The mitogen-activated protein kinases (*MAPKs*, *MPKs*) and *WRKY* transcription factors cascades are known to play important roles in mediating the plant biotic stresses in tobacco [20, 43]. In the current research, we chose two MAP kinase genes (*NtMPK1* and *NtMPK3*) and two WRKY transcription factors (*NtWRKY1* and *NtWRKY2*) to detect the crosstalk signal pathway. We found that these genes all showed similar induced expression patterns in tobacco seedlings under Cd stress and MeJA treatment, which simulates a pathogen invasion and biotic stress in tobacco. Although there should be multiple stress pathways regulated by MAPKs and WRKYs, our research emphasized the importance of how members of these two families might mediate the crosstalk between Cd stress and biotic stress in tobacco.

Reports have also indicated that Cd stress can disturb the balance of water homeostasis in plants and cause induced expression of dehydration-related genes [23, 44]. The *DREB* subfamily belongs to ABA-independent *AP2/ERF* (*APETALA2/ethylene-responsive element-binding*) transcription factors, widely involved in many regulatory roles in response to abiotic stresses, including drought/dehydration [45]. Aquaporin is a protein channel in the plasma membrane that facilitates water movement across the membrane, and also plays an important role in the response to drought stress [46]. In the current research, we found that two *DREB transcript factors* (*NtDREB1* and *NtDREB2*) and two *aquaporins* (*NtPIP1;1* and *NtPIP2;1*) showed similar induced expression patterns under Cd and mannitol stresses, which further proves that there was crosstalk between the Cd stress signal pathway and drought/dehydration.

**Fig 10:**
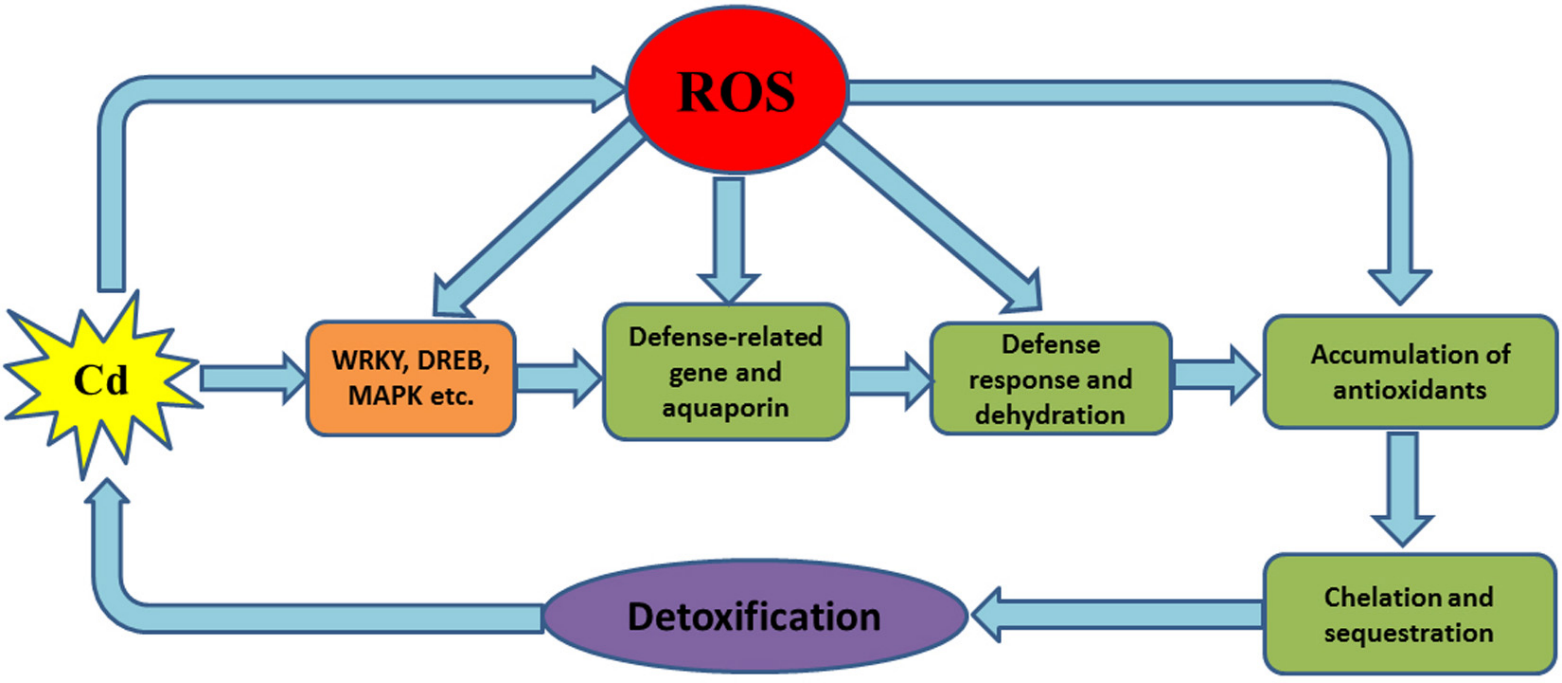
Schematic diagram illustrating the Cd-mediated signaling transduction by ROS accumulation in plant cells.

We have shown that all of the 24 Cd-tolerant candidate genes are involved in resistance to oxidative stress in yeast mutant *yap1Δ* and *skn7Δ* (Figs 4 and 5), and ROS accumulation seems to be a key point in the response of plant cells to many stresses [42], including heavy metal stresses. The transgenic yeast strains showed different accumulation of Cd, and overexpressing Cd-tolerant genes in yeast also conferred enhanced tolerance to improved oxidative stress tolerance. This, in combination with the genes’ expression analysis results under different stresses and hormones in tobacco, indicates that these Cd-tolerant candidate genes might participate in responses to different stress signaling pathways and play different roles in the process of Cd detoxification. Based on these results, we proposed the following working model for Cd toxicity and core function of ROS in plants (Fig 10): beside the direct substitution of essential mineral elements for plant growth (such as Zn, Ca, Fe, Mg, Mn, *etc*.), the ROS accumulation caused by Cd in plant cells can stimulate different signaling pathways and activate anti-oxidative system in the plant. This is accompanied by accumulation of metal-chelating protein (such as cysteine-rich protein) and transporters, and subsequently the Cd detoxification in plants is conducted. Therefore, to withstand Cd toxicity, some tobacco Cd-tolerant genes might augment ROS scavenging capacity indirectly and eventually enhance plant tolerance to adverse stresses. Further detailed analysis of the transgenic plants will provide more evidence for this hypothesis.

### Cysteine-rich proteins or peptides play pivotal roles in Cd detoxification

In recent decades, extensive studies have illustrated the mechanisms underlying Cd detoxification and tolerance in plants [15, 47] and the Cd tolerance-related genes that regulate the adaptive response of several plants to Cd contaminated soil have been identified [40]. It is well known that MTs play a major role in heavy metal detoxification. MTs belong to cysteine-rich proteins with a low molecular weight and can bind metal ions by disulfide bonds of highly conserved cysteine residues. *MTs* are induced in response to heavy metal stresses [27]. In our current study, a total of five tobacco *MTs* and one strain of *MT-like* cDNA were isolated by the library screening approach, and showed the strongest tolerance and largest proportion of the entire Cd-tolerant candidate gene pool (S3 Table). This indicates that *MTs* play an important role in Cd detoxification in tobacco plants.

In addition, some other cysteine-rich protein cDNAs, such as *NtSnakin-2* (*T9*), *NtRAR1* (*T17*) and *PLAC8 family member* (*T19*) were also isolated by the cDNA library screening in our study. Snakin/GASA proteins usually contain a clear C-terminal region of approximately 60 amino acids containing 12 cysteine residues in conserved positions known as the GASA domain [30]. Recent research has shown that one of snakin proteins in *Arabidopsis*, AtGASA5, is a redox-active metalloprotein and can bind Fe atoms in vitro, but not other elements (e.g., Ag, Al, B, Ba, Ca) [48]. Transcriptomic screening work in a Cd hyperaccumulating plant, *Viola baoshanensis* [49], showed that two *snakin* cDNAs (*hhcrp00004* and *hhcrp00005*) were induced by Cd treatment. Although there is little evidence for plant *snakin* being involved in heavy metal response, our library screening work and expression analyses here first presented and further confirmed that Snakin-2 might be an important Cd detoxification cysteine-rich protein in tobacco.

Plant RAR1 is a Zn binding protein, and often contains two cysteine- and histidine-rich domains (CHORDs) at the N- and C- terminus separately and a cysteine- and histidine-containing motif (CCCH motif) located between the two CHORDs. We speculate that Cd replaces Zn and binds to the tobacco RAR1 (*T17*) protein, which decreases the intracellular concentration of free Cd in the plant cells, just like in yeast cells. One of the tobacco *PLAC8 family members* (*T19*), a homology of *AtPCR1* and *AtPCR2* (*Arabidopsis* *P**lant* *C**admium* *R**esistance*), also encodes a small cysteine-rich membrane protein. AtPCR1 and AtPCR2 play important roles in transporting heavy metals such as Cd or Zn [50, 51], and here we supposed that this tobacco PLAC8 family member protein (*T19*) and thus might detoxify cellular Cd through similar mechanisms.

### Cadmium accumulation and tolerance in cells is concerned chiefly with chelation, secretion and compartmentalization

Generally speaking, the detoxification effect of cellular Cd mainly depends on decreasing the free Cd concentration in the cytoplasm. Because the higher plant is a type of multicellular organism, Cd translocation between different cells is mainly mediated by transporters or some secretory proteins. In our current study, we checked Cd content in yeast by expressing different candidate genes for tobacco Cd tolerance. Our results showed that although these genes can all increase the tolerance of *ycf1Δ* to Cd, the effects generated by different genes were not the same. Those genes whose expression increased the accumulation in yeast under low level Cd treatment (15 μM), such as *MTs* (*T10*, *T11*, *T15*, *T60* and *T89*), *RAR1* (*T17*), *glycine-rich protein* (*T22*) and *ubiquitin member* (*T53*), might encode metal chelating proteins, and their detoxification effect is mainly mediated by chelation or cellular compartmentalization. However, other candidate tolerant proteins, such as NtSnakin-2 (*T9*), Copper transporter (*T18*), Basic PR (*T24*), Unknown protein1 (*T39*), *MT-like* (*T40*), and Calreticulin (*T57*), might cause Cd detoxification by secretion.

## Conclusions

In summary, we have determined a series of candidate Cd tolerant/detoxification functional genes, and laid a good foundation for elucidating the molecular mechanisms of tobacco responding to Cd. Our study has provided the following new information: (1) the anti-oxidation system of tobacco plays an important role in Cd tolerant/detoxification; (2) plant response of Cd stresses signaling pathways are involved in cross-talks with other biological and abiotic stress signaling pathways, mainly through participation in oxidative stress responses. Further research will be performed to identify the biological functions of some candidate Cd-tolerant genes in plants based on transgenic techniques.

## Supporting Information

S1 Fig The oxidation resistance test of tobacco cDNA (24 candidate Cd detoxification cDNA in total) overexpression in yeast mutant *yap1Δ*. Yeast cultures were adjusted to OD_600_ = 2, 0.2, 0.02, 0.002 and 2 μl serial dilutions (from left to right in each panel) were spotted on SD medium without (YNB) or with different CdCl_2_ (0.02 mM and 0.05 mM) concentrations. As a negative control, the mutant strain *yap1Δ* was transformed with the empty vector pYES260 (EV). As a positive control, wild type yeast BY4741 (WT) was transformed with the empty vector pYES260 (EV). Plates were incubated for 6 days at 30°C.
(TIF)

S2 Fig Gene expression analyses of 24 candidate Cd detoxification genes in tobacco seedling roots and leaves under methyl viologen (100 μM) or mannitol stress (300 mM). Results represent means ± standard error of three biological replicates. Error bars: “a” indicates no significant differences (*P* > 0.05); “b” indicates a significant difference (0.01 < *P* < 0.05, compared with CK); and “c” indicates a highly significant difference (*P* < 0.01, compared with CK).
(TIF)

S1 Table Primers for amplifying and constructing yeast express recombinant plasmids of tobacco Cd-tolerant genes. (DOCX)

S2 Table Primers for real time RT-PCR detection of candidate gene responses to Cd, MeJA and mannitol. (DOCX)

S3 Table Sequence analyses of the tobacco root cDNA library functional screening with the yeast Cd-sensitive mutant *ycf1Δ*. (DOCX)
