# Supplementary figures and images for: Systematic Isolation and Characterization of Cadmium Tolerant Genes in Tobacco: A cDNA Library Construction and Screening Approach
Source: PLoS One. 2016 Aug 31;11(8):e0161147. doi: 10.1371/journal.pone.0161147 (PMC5007098; doi:10.1371/journal.pone.0161147)

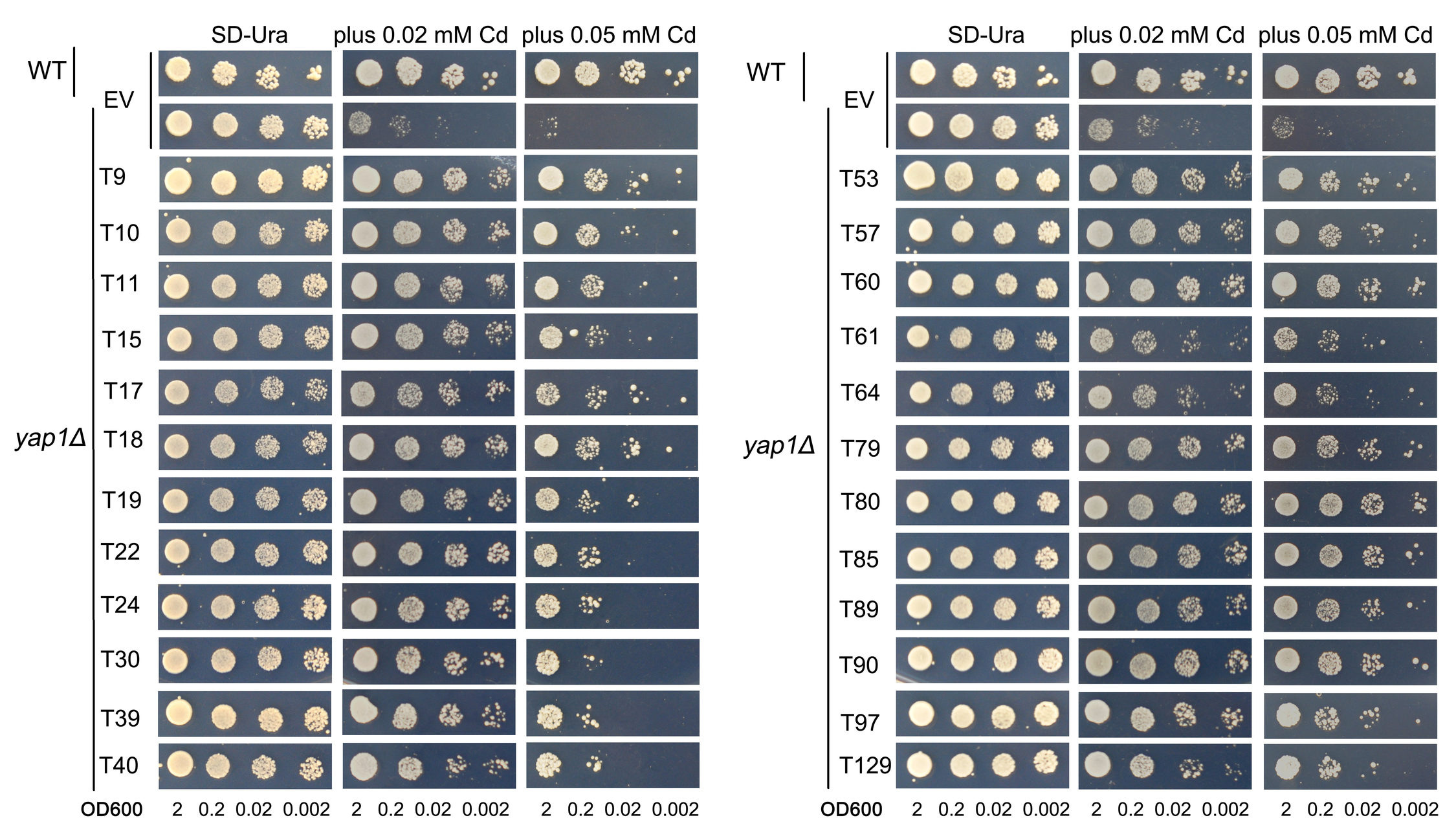

Supplement: S1 Fig — Yeast cultures were adjusted to OD600 = 2, 0.2, 0.02, 0.002 and 2 μl serial dilutions (from left to right in each panel) were spotted on SD medium without (YNB) or with different CdCl2 (0.02 mM and 0.05 mM) concentrations. As a negative control, the mutant strain yap1Δ was transformed with the empty vector pYES260 (EV). As a positive control, wild type yeast BY4741 (WT) was transformed with the empty vector pYES260 (EV). Plates were incubated for 6 days at 30°C. (TIF) [file pone.0161147.s001.tif]

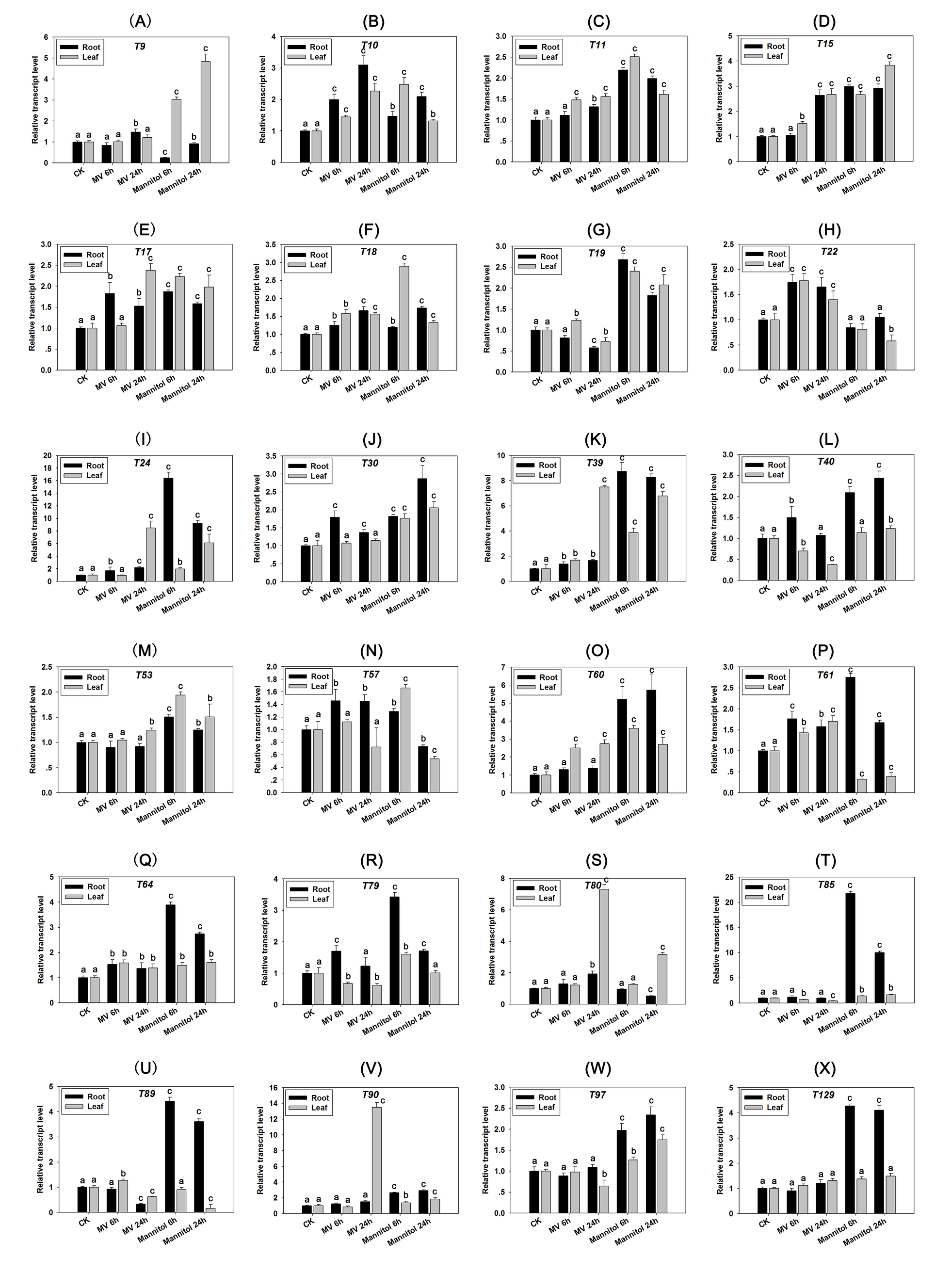

Supplement: S2 Fig — Results represent means ± standard error of three biological replicates. Error bars: “a” indicates no significant differences (P > 0.05); “b” indicates a significant difference (0.01 < P < 0.05, compared with CK); and “c” indicates a highly significant difference (P < 0.01, compared with CK). (TIF) [file pone.0161147.s002.tif]
