# Supplementary material for: Systematic Isolation and Characterization of Cadmium Tolerant Genes in Tobacco: A cDNA Library Construction and Screening Approach
Source: PLoS One. 2016 Aug 31;11(8):e0161147. doi: 10.1371/journal.pone.0161147 (PMC5007098; doi:10.1371/journal.pone.0161147)
Supplement: S1 Table — (DOCX) [file pone.0161147.s003.docx]

**Supporting information table 1: Primers for amplify and construct yeast express recombinant plasmids of Cd-tolerant genes in tobacco.**

| Primer | Sequence（5' → 3'） |
| --- | --- |
| T9YEF  T9YER | TTTCAGGGCGCCATGGCCATTTCTAAAACTCTCTTTG |
|  | CGTTACTAGTGGATCTTAAGGGCATTTGCGTCTGTTG |
| T10YEF | TTTCAGGGCGCCATGTCTTGTTGCGGAGGAAGTTG |
| T10YER | CGTTACTAGTGGATCTTAACAATTGCAAGGGTCAC |
| T11YEF | TTTCAGGGCGCCATGTCTTGCTGTGGAGGAAGCTG |
| T11YER | CGTTACTAGTGGATCTCAGCAGTTGCAAGGGTCAC |
| T15YEF | TTTCAGGGCGCCATGTCTTGCTGTGGAGGAAACTG |
| T15YER | CGTTACTAGTGGATCTTAGCCAATAAAGGCAAACA |
| T17EF | TTTCAGGGCGCCATGGAGAGACTTCGATGCCAGAG |
| T17ER | CGTTACTAGTGGATCTTAGGACGCTGGGTTGGCATTG |
| T18YEF | TTTCAGGGCGCCATGTCTCAGACCGTTGTTCTCAAG |
| T18YER | CGTTACTAGTGGATCTCAGGCTGCAGCAACAGCTTC |
| T19YEF | TTTCAGGGCGCCATGGCCAATAACGAAGAATCAAAC |
| T19YER | CGTTACTAGTGGATCTTAGCGGCCCATGGTCTGGTC |
| T22YEF | TTTCAGGGCGCCATGGCTGAGGAGAGTGGACAGAG |
| T22YER | CGTTACTAGTGGATCTCAACGACCTCCACTGGTAC |
| T24YEF | TTTCAGGGCGCCATGGGTGCTTGTTTGTCTTCCTC |
| T24YER | CGTTACTAGTGGATCTTAAGTAAACAAGAGAACGG |
| T30YEF | TTTCAGGGCGCCATGTCGATCTTTGAGTACAATGG |
| T30YER | CGTTACTAGTGGATCCTAGTCCATCCTTCCCTTCAAG |
| T39YEF | TTTCAGGGCGCCATGGGAAACTTTACTTCATGTAC |
| T39YER | CGTTACTAGTGGATCTCATCTAGAACAAACTGGTTC |
| T40YEF | TTTCAGGGCGCCATGTTTTCCAAAACTAACCTTTT |
| T40YER | CGTTACTAGTGGATCTTAGTCATGACATTTAGGAC |
| T53YEF | TTTCAGGGCGCCATGATAGAGGTGGTGTTGAACGATC |
| T53YER | CGTTACTAGTGGATCTTAGTTGTAGTAGAGCTCAAGG |
| T57YEF | TTTCAGGGCGCCATGGCTACTCAACGAAGGGCAAACC |
| T57YER | CGTTACTAGTGGATCTTACAGTTCATCATGCGCTTC |
| T60YEF | TTTCAGGGCGCCATGTCTGGCTGCGGATCAAACTG |
| T60YER | CGTTACTAGTGGATCTTAACAGTTGCAAGGGTCAC |
| T61YEF | TTTCAGGGCGCCATGGGAAAGCTAAGTACACTTTTAT |
| T61YER | CGTTACTAGTGGATCTTATTCCTTGTCAACTGGGGATAC |
| T64YEF | TTTCAGGGCGCCATGAGGCTTTGTAAATTCACAGCTCTCTCTTCTC |
| T64YER | CGTTACTAGTGGATCTTACATAGTATCGACTAAAAG |
| T79YEF | TTTCAGGGCGCCATGGCAGAAGTGAAGTTGCTTGG |
| T79YER | CGTTACTAGTGGATCTTATTTGGGAGCTGCTGCAG |
| T80YEF | TTTCAGGGCGCCATGGATGGAAGTAGAATCAACAA |
| T80YER | CGTTACTAGTGGATCTTACTGTTTGTGCAACTCAAG |
| T85YEF | TTTCAGGGCGCCATGGATGAAATTCAAGTACCTCC |
| T85YER | CGTTACTAGTGGATCCTAAAATGGATATGTTGATAAT |
| T89YEF | TTTCAGGGCGCCATGAATTCAAACAACACTACTGA |
| T89YER | CGTTACTAGTGGATCCTAATTTTGGTTATGACATT |
| T90YEF | TTTCAGGGCGCCATGggagaaGAAAATAAGGTCATTCTACATG |
| T90YER | CGTTACTAGTGGATCTCAATTGGCCGGAGATTTGAAG |
| T97YEF | TTTCAGGGCGCCATGGGTAAGTGCTATCCCACCGTAAG |
| T97YER | CGTTACTAGTGGATCTTAAGCTTCAGCAAATCCCA |
| T129YEF | TTTCAGGGCGCCATGAGTTTCCCACAGGAACCACAC |
| T129YER | CGTTACTAGTGGATCTCACTTCACCATTTGTTTATG |
