# Supplementary material for: Systematic Isolation and Characterization of Cadmium Tolerant Genes in Tobacco: A cDNA Library Construction and Screening Approach
Source: PLoS One. 2016 Aug 31;11(8):e0161147. doi: 10.1371/journal.pone.0161147 (PMC5007098; doi:10.1371/journal.pone.0161147)
Supplement: S2 Table — (DOCX) [file pone.0161147.s004.docx]

**Supporting information table 2: Primers real time RT-PCR detection of candidate genes responding to cadmium, MeJA and mannitol.**

| Primers | Sequences（5' → 3'） |
| --- | --- |
| T9RTF  T9RTR | GCCGCTTATTCCTACCCGAA |
|  | GAAGTGCCAGGAGGAACACA |
| T10RTF | CGGAGGATGTGGGATGTACC |
| T10RTR | TTTGATCCGCATTTGCAGCC |
| T11RTF | GTGGGATGTACCCCGACTTG |
| T11RTR | ATCAGCAGTTGCAAGGGTCA |
| T15RTF | CACAACCGAGACTTTGGTGC |
| T15RTR | GGGGTTACACTTGCAGTCAGA |
| T17RTF | GCTGACAGATGAGCCCTGTT |
| T17RTR | CAAAGGCTTAAGCGCATCCC |
| T18RTF | GTCATGCCAAGGCTGTGTAG |
| T18RTR | CTTCTGGCTCCACATTTCCCT |
| T19RTF | CGTCGTGGGTGAGCTCAATA |
| T19RTR | GCATTGCTCCCGTAAAGCAC |
| T22RTF | GGGCTAAGGGAACCGTCAAG |
| T22RTR | AGCTAGGCTACGGAAACCCT |
| T24RTF | CCTCCTCCGCCTACGTGATA |
| T24RTR | TGCCATTTCAGACGCACTCA |
| T30RTF | TCAACGGCTAGTGTTCCGTC |
| T30RTR | CCTCGCATGCACCATACAGA |
| T39RTF | TTCAGCTTTGTCTGCCGATGA |
| T39RTR | TGACACTTCCGGCGAAATCT |
| T40RTF | GGTGCCGGAATCATCCGTAA |
| T40RTR | ACTCACACCAGCACAAGTACA |
| T53RTF | CTGCTCAGACTGGTACACGC |
| T53RTR | TTGTAGTAGAGCTCAAGGCCC |
| T57RTF | AGTGGGGACGTTGACCAAAA |
| T57RTR | GGTGGGAGAAGGTCCCAATC |
| T60RTF | TCATTCAGGGTGTTGCTCCC |
| T60RTR | AGAAAAGCCACGTCACACGA |
| T61RTF | CGCAAAACGTTAGGGCAACA |
| T61RTR | CTGTGGTCTGAGCTCCTGTG |
| T64RTF | GGCCTTGTGGAAGAGCCATA |
| T64RTR | TGACATCGTGGCAAGAAGGT |
| T79RTF | TGGCAAAGGAGAGGAGTCAG |
| T79RTR | CCCAGCCAAAATGCCATCAAA |
| T80RTF | GATTTGCACCATGCCCCTGT |
| T80RTR | CAAGCAGGCCTAGCCAAAAT |
| T85RTF | TCGAAAGGTTTCCTACGCCG |
| T85RTR | AGCTAAGAACTCAGCTGCCC |
| T89RTF | ACGTCTAAGGCAACTGCTCC |
| T89RTR | TGCCGGCGATATTTTGGTCA |
| T90RTF | AACCTCGACTCTTGCCTGAAG |
| T90RTR | TTCCATCTTCCAGCACGTTCA |
| T97RTF | ACTCTCTGGTGGCCATACCT |
| T97RTR | GTGAGCCTCAGCATAGTCCG |
| T129RTF | TGCGGCTTACACAACTTGGA |
| T129RTR | TCAGTGATTCGGTGAGGCTG |
| NtMPK1RTF | TCTTTGATCCAAGCAGGCGT |
| NtMPK1RTR | CGGATTCCCTCCAGATGAGC |
| NtMPK3RTF | GGCCTCGTATGTGCTGCTAT |
| NtMPK3RTR | CCTCTTCCTTTGGCGGTCTT |
| NtWRKY1RTF | TTCTTTTGGCGACGAGGACA |
| NtWRKY1RTR | ATAGCTTCCACTACCGCGTG |
| NtWRKY2RTF | ACAACAATGGTGCCAATGCC |
| NtWRKY2RTR | GCTCGTCCTTTGCTCTGGAA |
| NtDREB1RTF | GAGACAACATGCCCCAAGGA |
| NtDREB1RTR | CGGCAAAAGCATACCTTCCG |
| NtDREB2RTF | AGTTTCGCGAGACACGTCAT |
| NtDREB2RTR | AATATCCTTAGCGTCGGCGG |
| NtPIP1;1RTF | ACCACCACCAGCACCATTAT |
| NtPIP1;1RTR | ACCAAAAGCCCAAGCAACAC |
| NtPIP2;1RTF | GGGAAGGATTACGTGGACCC |
| NtPIP2;1RTR | CACCAGAGATACCGGCAGT |
| NtL25RTF | CCCCTCACCACAGAGTCTGC |
| NtL25RTR | AAGGGTGTTGTTGTCCTCAATCTT |

Accession number in Genbank: NtDREB1 (EU727155.1); NtDREB2 (EU727156.1); NtMPK1 (AB212070.1); NtMPK3 (JX076821); NtWRKY1 (AB022693.1); NtWRKY2 (AB020590.1); NtPIP1;1 (AF440271.1); NtPIP2;1 (AF440272.1)
