# Supplementary material for: Systematic Isolation and Characterization of Cadmium Tolerant Genes in Tobacco: A cDNA Library Construction and Screening Approach
Source: PLoS One. 2016 Aug 31;11(8):e0161147. doi: 10.1371/journal.pone.0161147 (PMC5007098; doi:10.1371/journal.pone.0161147)
Supplement: S3 Table — (DOCX) [file pone.0161147.s005.docx]

**Supporting information table 3. Sequence analyses of the tobacco root cDNA library functional screening with Cd yeast sensitive mutant *Δycf1*. F: full-length cDNA, P: partial cDNA.**

| Clone | GenBank match | | Name | Category | Note |
| --- | --- | --- | --- | --- | --- |
| T9 | | XM_009623044.1 | Snakin-2 | Stress and defense | F |
| T10, T12, T14, T36, T43 | | XM_009621032.1 | Metallothionein type 2 | Stress and defense | F |
| T11, T8, T13, T20, T54 | | XM_009629116.1 | Metallothionein type 2 | Stress and defense | F |
| T15 | | XM_009608450.1 | Metallothionein type 2 | Stress and defense | F |
| T17 | | XM_009626938.1 | Rar1, disease resistance protein, Cysteine and histidine-rich domain-containing protein. | Stress and defense | F |
| T18 | | XM_009801794.1 | Copper transporter | Transporter | F |
| T19 | | XM_009629259.1 | PLAC8 family | Development | F |
| T21 | | XM_009614111.1 | Histone H1 | Structural protein | F |
| T22 | | NM_001311181.1 | Glycine-rich protein | Structural protein | F |
| T23 | | XM_009610419.1 | RNA-binding protein 39 | Unclassified | P |
| T24, T29, T74 | | XM_009789771.1 | Basic pathogenesis-related protein | Stress and defense | F |
| T26, T27 | | XM_009630368.1 | 40S ribosomal protein S3a | Protein synthesis  /degradation | P |
| T28 | | XM_009600387.1 | Ribosomal protein L24-like protein | Protein synthesis  /degradation | F |
| T30 | | XM_009799919.1 | Peptidase isoform 1 | Stress and defense | F |
| T31 | | XM_009607344.1 | Transcription initiation factor TFIID subunit 13-like | Transcriptional regulation | F |
| T32 | | XM_009761238.1 | Uncharacterized | Unknown protein | P |
| T33 | | XM_009780920.1 | Uncharacterized | Unknown protein | F |
| T34 | | XM_009632285.1 | Uncharacterized | Unknown protein | P |
| T35 | | XM_009613982.1 | Glutathione S-transferase | Stress and defense | P |
| T37 | | XM_009790829.1 | Fructose-1, 6-biphosphate aldolase | Unclassified | P |
| T38 | | XM_009628220.1 | Proteasome subunit alpha type | Protein synthesis  /degradation | F |
| T39 | | XM_009759724.1 | Uncharacterized | Unknown protein | F |
| T40, T66 | | XM_009631103.1 | Metallothionein-like | Stress and defense | F |
| T53 | | XM_009782934.1 | Ubiquitin family | Protein synthesis  /degradation | F |
| T55 | | XM_009761961.1 | KH domain protein | Unclassified | P |
| T56 | | XM_009769617.1 | 60S ribosomal protein L5 | Protein synthesis  /degradation | F |
| T57 | | XM_009631851.1 | Calreticulin | Signal transduction | F |
| T59 | | XM_009777245.1 | P-rich protein EIG-I30 | Unclassified | P |
| T60, T65 | | XM_009765140.1 | Metallothionein type 2 | Stress and defense | F |
| T61 | | XM_009795289.1 | CBP20 | Stress and defense | F |
| T64, T62 | | XM_009777153.1 | Chitinase | Stress and defense | F |
| T63 | | AB248516.1 | Hypothetical protein | Unknown protein | F |
| T67 | | XM_009785930.1 | Transcription factor VIP1-like | Transcriptional regulation | P |
| T68 | | XM_009782707.1 | Vesicle-associated membrane protein | Transporter | F |
| T69 | | XM_009791590.1 | Transcription elongation factor SPT5 homolog 1 | Unclassified | P |
| T71 | | XM_009777070.1 | Actin depolymerizing factor 3 | Unclassified | F |
| T72 | | XM_009776529.1 | Cytochrome b561 and DOMON domain-containing protein | Unclassified | P |
| T73 | | XM_009761264.1 | Tryptophan--tRNA ligase | Unclassified | P |
| T75 | | XM_009775526.1 | 60S acidic ribosomal protein P3 | Protein synthesis  /degradation | P |
| T76 | | XM_009780219.1 | ABA receptor PYL4-like | Stress and defense | F |
| T77 | | XM_009770917.1 | Endoplasmic reticulum oxidoreductin-1-like | Stress and defense | P |
| T79 | | XM_009791409.1 | Glutathione S-transferase | Stress and defense | F |
| T80 | | XM_009790972.1 | Glutathione S-transferase | Stress and defense | F |
| T81 | | XM_009779569.1 | NtbZIP60 | Transcriptional regulation | P |
| T84 | | XM_009591009.1 | Protein cornichon homolog 4-like | Unclassified | F |
| T85 | | XM_009761772.1 | E3 ubiquitin-protein ligase PUB23-like | Protein synthesis  /degradation | F |
| T87 | | [XM_009608894.1](http://www.ncbi.nlm.nih.gov/nucleotide/697106735?report=genbank&log$=nucltop&blast_rank=1&RID=CAUHJ7HB016) | 40S ribosomal protein S15-like | Protein synthesis  /degradation | F |
| T89 | | M97362.1 | Metallothionein-like | Stress and defense | F |
| T90 | | XM_009773220.1 | Glutathione S-transferase | Stress and defense | F |
| T91 | | XM_009781858.1 | Uncharacterized protein At2g23090-like | Unknown protein | F |
| T92 | | XM_009602719.1 | Uncharacterized protein | Unknown protein | P |
| T93 | | XM_009792957.1 | Uncharacterized protein | Unknown protein | F |
| T95 | | XM_009603143.1 | Glycine-rich cell wall structural protein 1.8-like | Structural protein | F |
| T97 | | XM_009772965.1 | Ascorbate peroxidase | Stress and defense | F |
| T98 | | XM_009598253.1 | Putative nuclease HARBI1 | Unclassified | P |
| T99 | | XM_009769070.1 | Pentatricopeptide repeat-containing protein At3g13150-like | Transporter | P |
| T103 | | XM_009609755.1 | Pentatricopeptide repeat-containing protein At5g25630-like | Transporter | P |
| T104 | | XM_009773610.1 | DEAD-box ATP-dependent RNA helicase 56-like | Unclassified | P |
| T105 | | XM_009776754.1 | Mitogen-activated protein kinase kinase 2-like | Signal transduction | P |
| T106 | | AF352732.1 | Glutamate decarboxylase isozyme 1 | Unclassified | P |
| T108 | | XM_009772568.1 | Diacylglycerol kinase 7-like | Signal transduction | P |
| T109 | | XM_009790829.1 | Fructose-bisphosphate aldolase cytoplasmic isozyme | Unclassified | P |
| T111 | | CAA11153 | Short chain alcohol dehydrogenase | Unclassified | F |
| T113 | | XM_009777516.1 | Mitochondrial-processing peptidase subunit beta | Stress and defense | P |
| T114 | | X66942.1 | Pathogenesis-related protein 1b | Stress and defense | P |
| T115 | | XM_009632011.1 | Uncharacterized protein At4g28440-like | Unknown protein | P |
| T116 | | D26459.1 | mRNA for tumor-related protein | Unclassified | P |
| T117 | | XM_009792661.1 | ACT domain-containing protein ACR3 | Transporter | F |
| T118 | | XM_009761818.1 | Ethylene-responsive TF9-like | Transcriptional regulation | P |
| T119 | | XM_009761178.1 | Actin-101 | Structural protein | P |
| T120 | | XM_009785993.1 | Uncharacterized | Unknown protein | P |
| T122 | | XM_009622076.1 | Uncharacterized protein At1g15400-like | Unknown protein | F |
| T123 | | [XM_009804447.1](http://www.ncbi.nlm.nih.gov/nucleotide/698515742?report=genbank&log$=nucltop&blast_rank=1&RID=C8B61GMB014) | Dihydrolipoyllysine-residue succinyltransferase component | Unclassified | P |
| T124 | | AB121785.1 | Pathogenesis-related protein PR-5d | Stress and defense | P |
| T125 | | XM_009762213.1 | Ribonuclease 3-like protein 3 | Unclassified | P |
| T126 | | BA000042.1 | mitochondrial DNA | Unclassified | P |
| T127 | | HF675180.1 | HMA-A gene for heavy metal ATPase | Transporter | P |
| T128 | | XM_009804774.1 | 4-coumarate--CoA ligase-like 10 | Unclassified | F |
| T129 | | XM_009769585.1 | Bypass1 | Development | F |
| T130 | | XM_009590462.1 | F-box protein SKP2A-like | Unclassified | F |
| T132 | | XM_009796557.1 | Histone H2A | Structural protein | F |
| T134 | | XM_009792350.1 | Putative G3BP-like protein | Unclassified | F |
| T135 | | XM_009785609.1 | Actin-related protein 7 | Structural protein | P |
